# Correction: Shifting food web structure during dam removal—Disturbance and recovery during a major restoration action

**DOI:** 10.1371/journal.pone.0246517

**Published:** 2021-01-28

**Authors:** The PLOS ONE Staff

## Notice of Republication

This article was republished on November 19, 2020, to correct errors in the tables that were introduced during the typesetting process. The publisher apologizes for the errors. Please download this article again to view the correct version. The originally published, uncorrected article and the republished, corrected articles are provided here for reference.

## Supporting information

S1 File Originally published, uncorrected article. (PDF)

S2 File Republished, corrected article. (PDF)
