# Supplementary figures and images for: Shifting food web structure during dam removal—Disturbance and recovery during a major restoration action
Source: PLoS One. 2020 Sep 29;15(9):e0239198. doi: 10.1371/journal.pone.0239198 (PMC7523948; doi:10.1371/journal.pone.0239198)

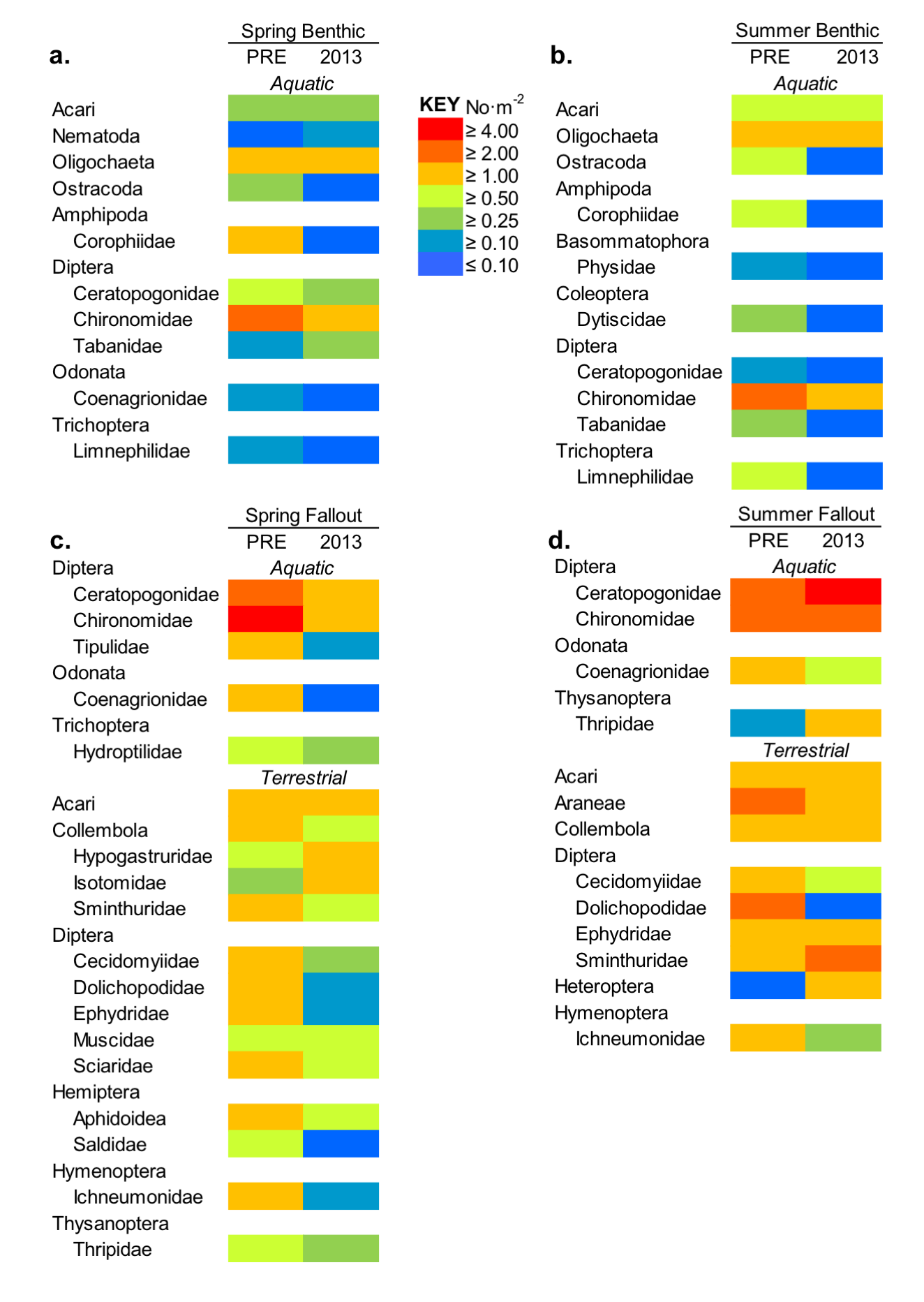

Supplement: S1 Fig — Taxa are shown for benthic grab (a,b) and fallout samples (c,d) that contributed most to differences between years in spring (a,c) and summer (b,d), based on the SIMPER routine in PRIMER (≥ 2% dissimilarity). Mean numerical density is fourth root transformed. (TIFF) [file pone.0239198.s001.tiff]

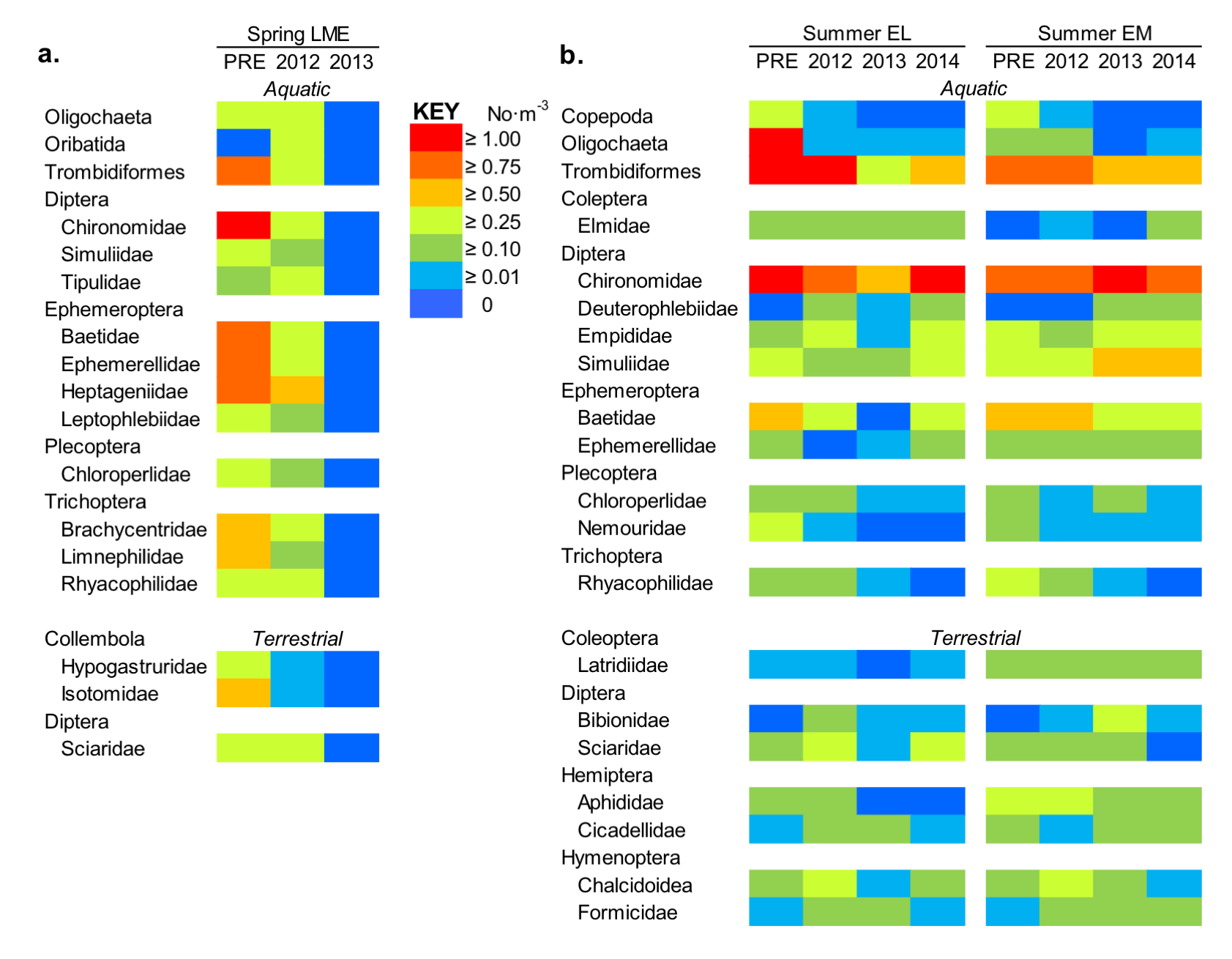

Supplement: S2 Fig — Taxa are shown that contributed the most to differences between years in (a) spring and (b) summer river drift, based on the SIMPER routine in PRIMER (≥ 2% dissimilarity). Mean numerical density by river section and year are square-root transformed. (TIFF) [file pone.0239198.s002.tiff]

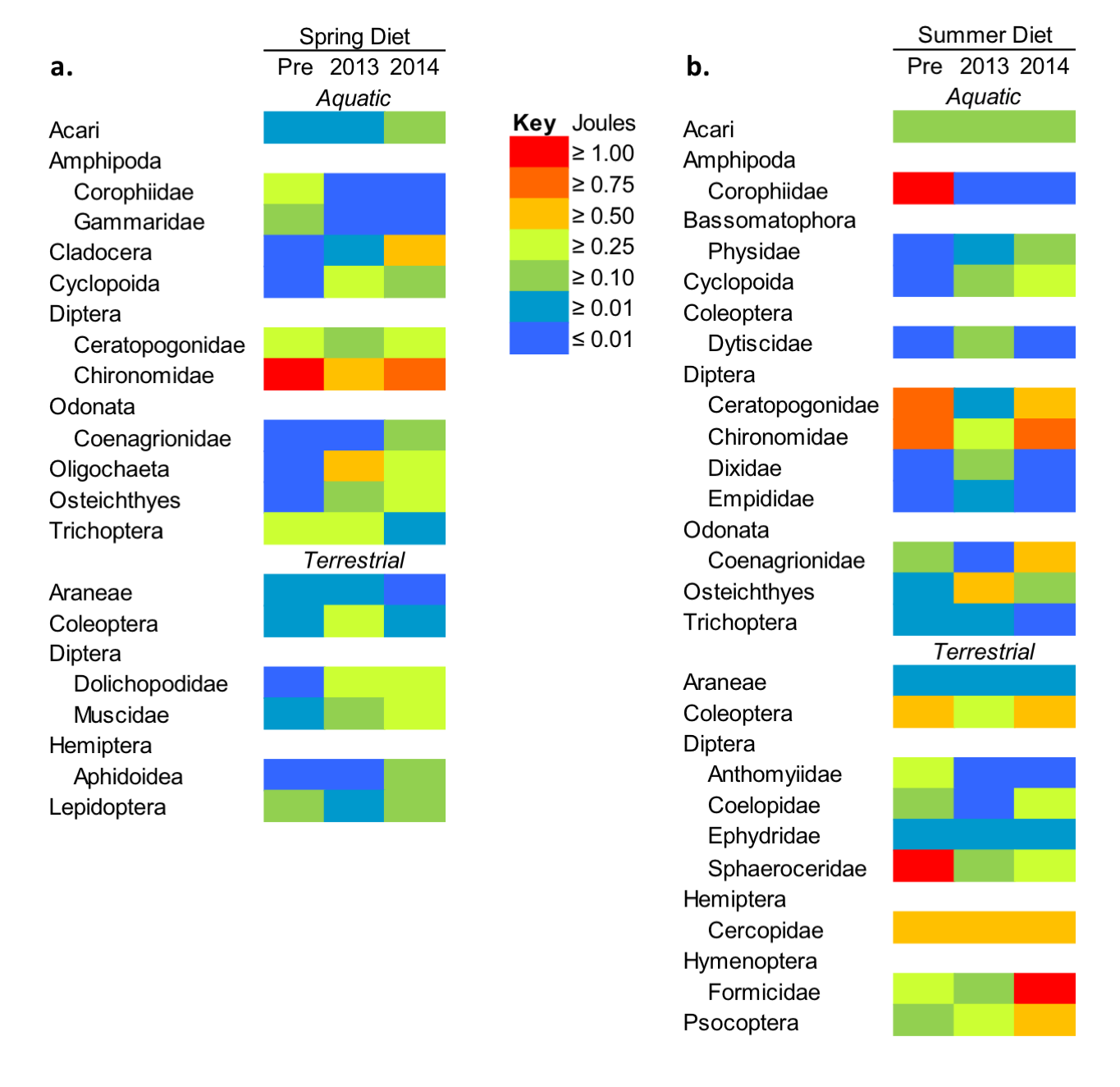

Supplement: S3 Fig — Taxa are shown that contributed the most to differences between years in (a) spring, and (b) summer estuary fish diet, based on the SIMPER routine in PRIMER (≥ 2% dissimilarity). Mean joules by season and year are fourth root transformed. (TIFF) [file pone.0239198.s003.tiff]

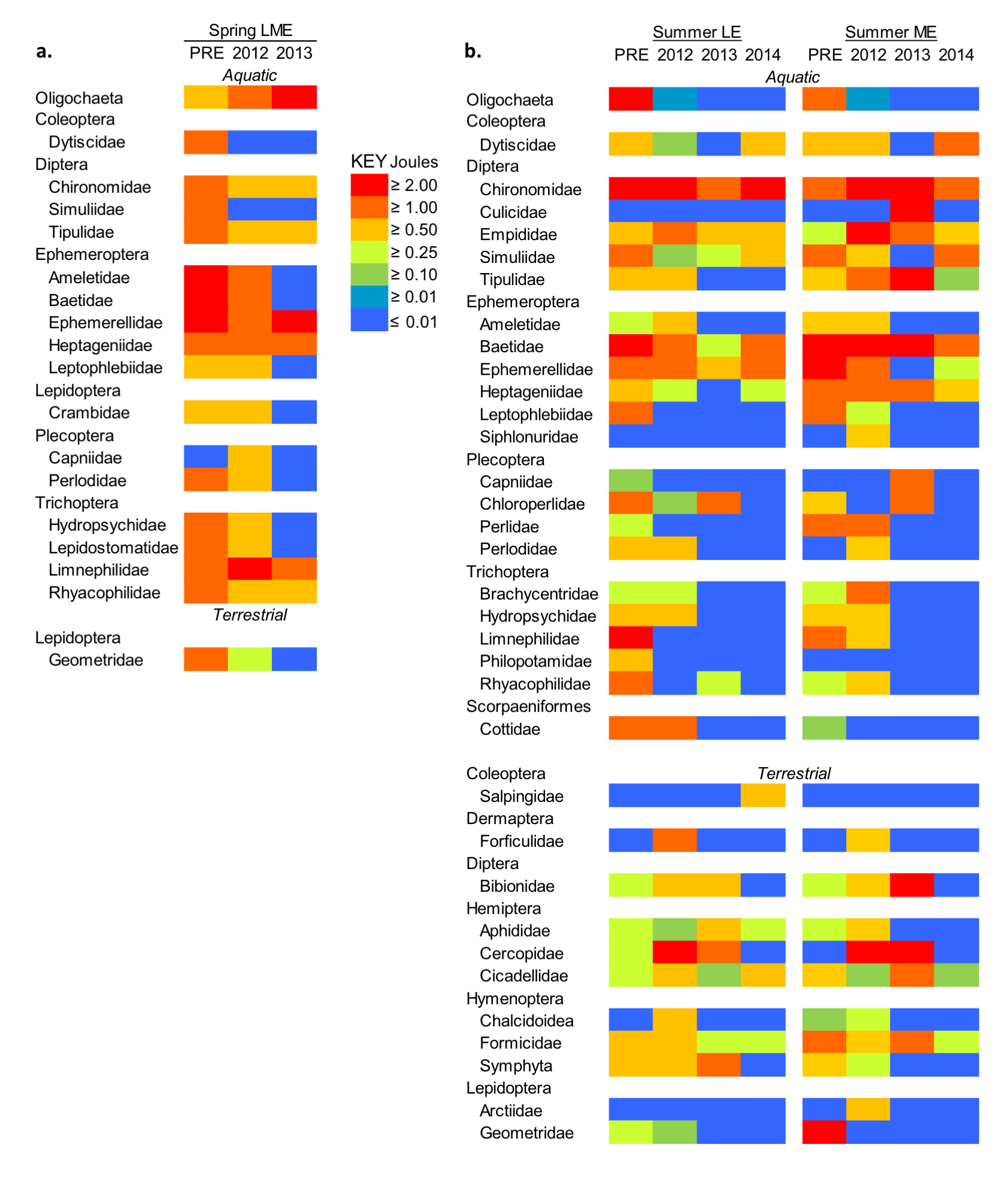

Supplement: S4 Fig — Taxa are shown that contributed the most to differences between years in a) spring, and b) summer estuary fish diet, based on the SIMPER routine in PRIMER (≥ 2% dissimilarity). Mean joules by season and year are fourth root transformed. (TIFF) [file pone.0239198.s004.tiff]
