# Supplementary material for: Shifting food web structure during dam removal—Disturbance and recovery during a major restoration action
Source: PLoS One. 2020 Sep 29;15(9):e0239198. doi: 10.1371/journal.pone.0239198 (PMC7523948; doi:10.1371/journal.pone.0239198)
Supplement: S1 Table — (PDF) [file pone.0239198.s005.pdf]

S1 Table. Aquatic-origin invertebrates collected in environmental samples during the study. Estuary invertebrate samples were collected by ponar grab, and river invertebrates by drift samples. EE = Elwha estuary, LE = lower Elwha, ME = middle Elwha, TR = tributaries.

Class	Order	Family	Genus	Species	EE	LE	ME	TR
Insecta	Coleoptera	Agabini						
Insecta	Coleoptera	Dytiscidae						
Insecta	Coleoptera	Dytiscidae	*Oreodytes*					
Insecta	Coleoptera	Dytiscidae	*Stictotarsus*					
Insecta	Coleoptera	Elmidae	*Heterlimnius*	*H. corpulentus*				
Insecta	Coleoptera	Elmidae	*Lara*					
Insecta	Coleoptera	Elmidae	*Narpus*	*N. concolor*				
Insecta	Coleoptera	Elmidae	*Optioservus*					
Insecta	Coleoptera	Elmidae	*Zaitzevia*					
Insecta	Coleoptera	Halipidae						
Insecta	Coleoptera	Hydrophilidae						
Insecta	Coleoptera	Hydrophilidae	*Anacaena*					
Insecta	Coleoptera	Hydroporinae						
Insecta	Diptera	Agromyzidae						
Insecta	Diptera	Anisopodidae	*Sylvicola*					
Insecta	Diptera	Anthomyiidae						
Insecta	Diptera	Asteiidae						
Insecta	Diptera	Athericidae	*Atherix*					
Insecta	Diptera	Blephariceridae						
Insecta	Diptera	Blephariceridae	*Agathon*					
Insecta	Diptera	Calliphoridae						
Insecta	Diptera	Cecidomyiidae						
Insecta	Diptera	Ceratopogonidae						
Insecta	Diptera	Ceratopogonidae	*Bezzia*					
Insecta	Diptera	Ceratopogonidae	*Ceratopogoninae*					
Insecta	Diptera	Ceratopogonidae	*Culicoides*					
Insecta	Diptera	Ceratopogonidae	*Forcipomyiinae*					
Insecta	Diptera	Chaoboridae						
Insecta	Diptera	Chironomidae	*Chironomini*					
Insecta	Diptera	Chironomidae	*Diamesinae*					
Insecta	Diptera	Chironomidae	*Orthocladiinae*					
Insecta	Diptera	Chironomidae	*Podonominae*					
Insecta	Diptera	Chironomidae	*Prodiamesinae*					
Insecta	Diptera	Chironomidae	*Tanypodinae*					
Insecta	Diptera	Chironomidae	*Tanytarsini*					
Insecta	Diptera	Chloropidae						
Insecta	Diptera	Coelopidae						
Insecta	Diptera	Culicidae	*Culex*					
Insecta	Diptera	Culicidae	*Culiseta*					
Insecta	Diptera	Deuterophlebiidae	*Deuterophlebia*					

Class	Order	Family	Genus	Species	EE	LE	ME	TR
Insecta	Diptera	Dixidae	*Dixa*		■	■	■	■
Insecta	Diptera	Dixidae	*Dixella*		■		■	■
Insecta	Diptera	Dixidae	*Meringodixa*				■	■
Insecta	Diptera	Dolichopodidae			■	■	■	■
Insecta	Diptera	Drosophilidae			■			
Insecta	Diptera	Empididae	*Chelifera*		■	■	■	
Insecta	Diptera	Empididae	*Clinocera*			■	■	■
Insecta	Diptera	Empididae	*Hemerodromia*		■			■
Insecta	Diptera	Empididae	*Hilara*		■			
Insecta	Diptera	Empididae	*Neoplasta*					■
Insecta	Diptera	Empididae	*Oreogeton*			■	■	■
Insecta	Diptera	Empididae	*Rhamphomyia*		■			
Insecta	Diptera	Empididae	*Trichoclinocera*				■	
Insecta	Diptera	Ephydridae			■	■		■
Insecta	Diptera	Heleomyzidae			■			
Insecta	Diptera	Hybotidae	*Platypalpus*		■			
Insecta	Diptera	Muscidae			■		■	■
Insecta	Diptera	Muscamorpha			■			
Insecta	Diptera	Mycetophilidae			■			
Insecta	Diptera	Nematocera			■			
Insecta	Diptera	Phoridae			■	■		■
Insecta	Diptera	Psychodidae			■	■	■	
Insecta	Diptera	Psychodidae	*Maruina*					■
Insecta	Diptera	Psychodidae	*Pericoma/Telmatoscopus*					■
Insecta	Diptera	Rhagionidae			■			
Insecta	Diptera	Rhagionidae	*Chrysopilus*		■			
Insecta	Diptera	Rhagionidae	*Rhagio*		■			
Insecta	Diptera	Scathophagidae			■			
Insecta	Diptera	Scatopsidae			■			
Insecta	Diptera	Sciaridae			■			
Insecta	Diptera	Sciomyzidae			■			
Insecta	Diptera	Simuliidae			■			
Insecta	Diptera	Simuliidae	*Helodon*					■
Insecta	Diptera	Simuliidae	*Prosimulium*				■	■
Insecta	Diptera	Simuliidae	*Simulium*			■	■	■
Insecta	Diptera	Stratiomyidae					■	
Insecta	Diptera	Syrphidae			■			
Insecta	Diptera	Tabanidae			■			■
Insecta	Diptera	Tabanidae	*Chrysops*		■			■
Insecta	Diptera	Tabanidae	*Hybomitra*		■			
Insecta	Diptera	Thaumaleidae						■
Insecta	Diptera	Tipulidae	*Antocha*	*A. monticola*		■	■	■
Insecta	Diptera	Tipulidae	*Dicranota*			■		■
Insecta	Diptera	Tipulidae	*Hesperoconopa*					■

Class	Order	Family	Genus	Species	EE	LE	ME	TR
Insecta	Diptera	Tipulidae	*Limnophila*					■
Insecta	Diptera	Tipulidae	*Limonia*					■
Insecta	Diptera	Tipulidae	*Ormosia*		■			
Insecta	Diptera	Tipulidae	*Rhabdomastix*		■			
Insecta	Diptera	Tipulidae	*Tipula*					■
Insecta	Ephemeroptera	Ameletidae	*Ameletus*			■	■	■
Insecta	Ephemeroptera	Baetidae	*Acentrella*	*A. turbida*	■	■	■	
Insecta	Ephemeroptera	Baetidae	*Baetis*	*B. alius*	■	■	■	■
Insecta	Ephemeroptera	Baetidae	*Baetis*	*B. bicaudatus*			■	■
Insecta	Ephemeroptera	Baetidae	*Baetis*	*B. Rhodani Gr.*		■	■	■
Insecta	Ephemeroptera	Baetidae	*Baetis*	*B. tricaudatus*		■	■	■
Insecta	Ephemeroptera	Baetidae	*Centroptilum*				■	■
Insecta	Ephemeroptera	Baetidae	*Diphetor*	*D. hageni*		■	■	■
Insecta	Ephemeroptera	Ephemerellidae	*Attenella*	*A. delantala*				■
Insecta	Ephemeroptera	Ephemerellidae	*Caudatella*					■
Insecta	Ephemeroptera	Ephemerellidae	*Caudatella*	*C. edmundsi*			■	
Insecta	Ephemeroptera	Ephemerellidae	*Drunella*	*D. coloradensis*		■		■
Insecta	Ephemeroptera	Ephemerellidae	*Drunella*	*D. doddsii*			■	■
Insecta	Ephemeroptera	Ephemerellidae	*Drunella*	*D. flavilinea*		■	■	■
Insecta	Ephemeroptera	Ephemerellidae	*Drunella*	*D. spinifera*				■
Insecta	Ephemeroptera	Ephemerellidae	*Ephemerella*	*E. excrucians*			■	■
Insecta	Ephemeroptera	Ephemerellidae	*Ephemerella*	*E. tibialis*		■	■	■
Insecta	Ephemeroptera	Ephemerellidae	*Timpanoga*	*T. hecuba*			■	
Insecta	Ephemeroptera	Ephemerellidae	*Matriella*	*M. teresa*				■
Insecta	Ephemeroptera	Heptageniidae	*Cinygmula*		■	■	■	■
Insecta	Ephemeroptera	Heptageniidae	*Epeorus*			■	■	■
Insecta	Ephemeroptera	Heptageniidae	*Ironodes*					■
Insecta	Ephemeroptera	Heptageniidae	*Rhithrogena*				■	■
Insecta	Ephemeroptera	Leptophlebiidae	*Paraleptophlebia*				■	■
Insecta	Ephemeroptera	Leptophlebiidae	*Paraleptophlebia*	*P. bicornuta*		■		
Insecta	Ephemeroptera	Siphlonuridae	*Siphlonurus*		■			
Insecta	Hemiptera	Corixidae			■	■		
Insecta	Hemiptera	Gerridae			■	■	■	■
Insecta	Hemiptera	Saldidae	*Saldula*				■	
Insecta	Lepidoptera						■	
Insecta	Lepidoptera	Crambidae				■	■	■
Insecta	Megaloptera	Sialidae	*Sialis*		■		■	■
Insecta	Odonata	Coenagrionidae	*Engallagma*		■			
Insecta	Odonata	Coenagrionidae	*Ischnura*		■			
Insecta	Odonata	Sympetrum			■			
Insecta	Plecoptera	Capniidae				■	■	■
Insecta	Plecoptera	Chloroperlidae			■			
Insecta	Plecoptera	Chloroperlidae	*Plumiperla*	*P. diversa*			■	
Insecta	Plecoptera	Chloroperlidae	*Suwallia*			■	■	

Class	Order	Family	Genus	Species	EE	LE	ME	TR
Insecta	Plecoptera	Chloroperlidae	*Sweltsa*		■	■	■	■
Insecta	Plecoptera	Leuctridae				■	■	
Insecta	Plecoptera	Leuctridae	*Despaxia*	*D. augusta*				■
Insecta	Plecoptera	Nemouridae	*Malenka*			■	■	■
Insecta	Plecoptera	Nemouridae	*Podmosta*				■	
Insecta	Plecoptera	Nemouridae	*Soyedina*				■	■
Insecta	Plecoptera	Nemouridae	*Visoka*	*V. cataractae*				■
Insecta	Plecoptera	Nemouridae	*Zapada*	*Z. cinctipes*		■	■	■
Insecta	Plecoptera	Nemouridae	*Zapada*	*Z. columbiana*			■	■
Insecta	Plecoptera	Nemouridae	*Zapada*	*Z. Oregonensis Gr.*			■	■
Insecta	Plecoptera	Peltoperlidae	*Yoraperla*					■
Insecta	Plecoptera	Perlidae	*Calineuria*			■		
Insecta	Plecoptera	Perlidae	*Calineuria*	*C. californica*				■
Insecta	Plecoptera	Perlidae	*Hesperoperla*	*H. pacifica*			■	■
Insecta	Plecoptera	Perlodidae	*Kogotus*			■	■	■
Insecta	Plecoptera	Perlodidae	*Skwala*			■	■	■
Insecta	Plecoptera	Pteronarcyidae	*Pteronarcella*					■
Insecta	Plecoptera	Pteronarcyidae	*Pteronarcys*	*P. californica*				■
Insecta	Plecoptera	Taeniopterygidae					■	
Insecta	Trichoptera	Apataniidae					■	
Insecta	Trichoptera	Brachycentridae	*Amiocentrus*	*A. aspilus*		■	■	
Insecta	Trichoptera	Brachycentridae	*Brachycentrus*	*B. americanus*		■	■	■
Insecta	Trichoptera	Brachycentridae	*Brachycentrus*				■	■
Insecta	Trichoptera	Brachycentridae	*Micrasema*			■	■	■
Insecta	Trichoptera	Glossosomatidae			■			
Insecta	Trichoptera	Glossosomatidae	*Anagapetus*					■
Insecta	Trichoptera	Glossosomatidae	*Glossosoma*				■	■
Insecta	Trichoptera	Hydropsychidae	*Cheumatopsyche*					■
Insecta	Trichoptera	Hydropsychidae	*Hydropsyche*		■	■	■	■
Insecta	Trichoptera	Hydropsychidae	*Parapsyche*					■
Insecta	Trichoptera	Hydroptilidae	*Hydroptila*		■		■	
Insecta	Trichoptera	Hydroptilidae	*Ochrotrichia*		■		■	
Insecta	Trichoptera	Hydroptilidae	*Stactobiella*			■	■	
Insecta	Trichoptera	Integripalpia			■			
Insecta	Trichoptera	Lepidostomatidae	*Lepidostoma*		■	■	■	■
Insecta	Trichoptera	Leptoceridae	*Mystacides*					■
Insecta	Trichoptera	Limnephilidae			■			
Insecta	Trichoptera	Limnephilidae	*Dicosmoecus*	*D. atripes*				■
Insecta	Trichoptera	Limnephilidae	*Dicosmoecus*	*D. gilvipes*		■	■	
Insecta	Trichoptera	Limnephilidae	*Ecclisomyia*				■	■
Insecta	Trichoptera	Limnephilidae	*Eocosmoecus*				■	
Insecta	Trichoptera	Limnephilidae	*Onocosmoecus*	*O. unicolor*				■
Insecta	Trichoptera	Limnephilidae	*Psychoglypha*		■		■	■
Insecta	Trichoptera	Philopotamidae	*Wormaldia*			■		■

Class	Order	Family	Genus	Species	EE	LE	ME	TR
Insecta	Trichoptera	Polycentropodidae	*Polycentropus*			■		■
Insecta	Trichoptera	Rhyacophilidae			■			
Insecta	Trichoptera	Rhyacophilidae	*Rhyacophila*	*R. Angelita Gr.*		■	■	■
Insecta	Trichoptera	Rhyacophilidae	*Rhyacophila*	*R. arnaudi*			■	
Insecta	Trichoptera	Rhyacophilidae	*Rhyacophila*	*R. Betteni Gr.*		■	■	■
Insecta	Trichoptera	Rhyacophilidae	*Rhyacophila*	*R. Brunnea/Vemna Gr.*		■	■	■
Insecta	Trichoptera	Rhyacophilidae	*Rhyacophila*	*R. Hyalinata Gr.*		■	■	■
Insecta	Trichoptera	Rhyacophilidae	*Rhyacophila*	*R. narvae*		■	■	■
Insecta	Trichoptera	Rhyacophilidae	*Rhyacophila*	*R. Nevadensis Gr.*		■		■
Insecta	Trichoptera	Rhyacophilidae	*Rhyacophila*	*R. Vagrita Gr.*				■
Insecta	Trichoptera	Rhyacophilidae	*Rhyacophila*	*R. Verrula Gr.*				■
Insecta	Trichoptera	Rhyacophilidae	*Rhyacophila*	*R. Vofixa Gr.*				■
Insecta	Trichoptera	Uenoidae	*Neophylax*	*N. splendens*				■
Insecta	Trichoptera	Uenoidae	*Oligophlebodes*					■
Acari	Oribatida					■	■	■
Acari	Trombidiformes	Hydrovolziidae	*Hydrovolzia*					■
Acari	Trombidiformes	Hygrobatidae	*Atractides*			■	■	■
Acari	Trombidiformes	Hygrobatidae	*Hygrobates*			■	■	■
Acari	Trombidiformes	Lebertiidae	*Estelloxus*				■	
Acari	Trombidiformes	Lebertiidae	*Lebertia*			■	■	■
Acari	Trombidiformes	Limnocharidae	*Limnochares*				■	■
Acari	Trombidiformes	Mideopsidae	*Mideopsis*			■		■
Acari	Trombidiformes	Oxidae	*Frontipoda*				■	■
Acari	Trombidiformes	Oxidae	*Oxus*			■	■	■
Acari	Trombidiformes	Protziidae	*Protzia*			■		■
Acari	Trombidiformes	Sperchonidae	*Sperchon*			■	■	■
Acari	Trombidiformes	Sperchonidae	*Sperchonopsis*			■	■	■
Acari	Trombidiformes	Torrenticolidae	*Testudacarus*				■	
Acari	Trombidiformes	Torrenticolidae	*Torrenticola*			■	■	■
Acari	Trombidiformes	Unionicolidae	*Unionicola*			■		■
Arachnida	Araneae				■			
Arachnida	Trombidiformes	Hydryphantidae	*Panisopsis*					■
Bivalvia	Veneroida	Sphaeriidae				■		
Clitellata	Branchiobdellida							■
Crustacea	Cladocera					■	■	■
Crustacea	Copepoda							■
Crustacea	Copepoda	Cyclopoida			■	■	■	
Crustacea	Isopoda				■			
Crustacea	Ostracoda				■		■	
Crustacea	Ostracoda	Podocopida			■	■		■
Gastropoda	Basommatophora				■			
Gastropoda	Basommatophora	Lymnaeidae			■			
Gastropoda	Basommatophora	Physidae			■			
Gastropoda	Basommatophora	Physidae	*Physa*			■	■	■

**Class**	**Order**	**Family**	**Genus**	**Species**	**EE**	**LE**	**ME**	**TR**
Gastropoda	Basommatophora	Planorbidae				■		
Gastropoda	Basommatophora	Planorbidae	*Menetus*				■	
Gastropoda	Neotaenioglossa	Hydrobiidae						■
Hexanauplia	Harpacticoida				■			
Hirudinea					■			
Hydrozoa						■	■	■
Malacostraca	Amphipoda	Corophiidae	*Corophium*		■			
Malacostraca	Amphipoda	Corophiidae	*Paracorophium*		■			
Malacostraca	Amphipoda	Crangonyctidae	*Crangonyx*					■
Malacostraca	Amphipoda	Gammaridae	*Gammarus*		■			
Malacostraca	Amphipoda	Hyalellidae	*Hyalella*					■
Oligochaeta					■	■	■	■
Nemata						■	■	■
Nematoda					■		■	■
Nematomorpha						■	■	■
Turbellaria	Tricladida	Planariidae	*Polycelis*	*P. coronata*		■	■	■
