# Supplementary material for: Shifting food web structure during dam removal—Disturbance and recovery during a major restoration action
Source: PLoS One. 2020 Sep 29;15(9):e0239198. doi: 10.1371/journal.pone.0239198 (PMC7523948; doi:10.1371/journal.pone.0239198)
Supplement: S2 Table — (PDF) [file pone.0239198.s006.pdf]

S2 Table. Terrestrial-origin invertebrates collected in environmental samples during the study. Estuary invertebrates were collected by fallout traps, and river invertebrates by drift samples. EE = Elwha estuary, LE = lower Elwha, ME = middle Elwha, TR = tributaries.

**Class**	**Order**	**Family**	**Genus**	**EE**	**LE**	**ME**	**TR**
Insecta	Coleoptera	Anobiidae	*Hemicoelus*	■	■		
Insecta	Coleoptera	Cantharidae		■	■	■	■
Insecta	Coleoptera	Carabidae		■	■	■	■
Insecta	Coleoptera	Cerambycidae		■	■		
Insecta	Coleoptera	Cerylonidae					■
Insecta	Coleoptera	Chrysomelidae		■		■	■
Insecta	Coleoptera	Ciidae		■			
Insecta	Coleoptera	Clambidae					
Insecta	Coleoptera	Coccinellidae		■		■	■
Insecta	Coleoptera	Coccinellidae	*Psyllobora*		■		
Insecta	Coleoptera	Curculionidae			■	■	■
Insecta	Coleoptera	Elateridae			■	■	■
Insecta	Coleoptera	Erotylidae					■
Insecta	Coleoptera	Hydrophilidae		■	■		
Insecta	Coleoptera	Lampyridae	*Ellychnia*	■			
Insecta	Coleoptera	Latridiidae		■			
Insecta	Coleoptera	Latridiidae	*Melanophthalma*		■	■	■
Insecta	Coleoptera	Leiodidae		■			
Insecta	Coleoptera	Melandryidae				■	
Insecta	Coleoptera	Melyridae			■		
Insecta	Coleoptera	Mordellidae				■	■
Insecta	Coleoptera	Ptiliidae		■	■		■
Insecta	Coleoptera	Salpingidae	*Rhinosimus*			■	
Insecta	Coleoptera	Scolytinae		■		■	■
Insecta	Coleoptera	Scraptiidae		■		■	
Insecta	Coleoptera	Silvanidae					■
Insecta	Coleoptera	Staphylinidae		■	■	■	■
Insecta	Coleoptera	Tenebrionidae					■
Insecta	Coleoptera	Throscidae		■	■		
Insecta	Dermaptera			■			
Insecta	Dermaptera	Forficulidae	*Forficula*	■			
Insecta	Diptera	Anthomyiidae		■			■
Insecta	Diptera	Asilidae			■	■	
Insecta	Diptera	Bibionidae			■	■	■
Insecta	Diptera	Cecidomyiidae		■	■	■	■
Insecta	Diptera	Empididae		■			■
Insecta	Diptera	Empididae	*Empis*			■	
Insecta	Diptera	Empididae	*Metachela*	■			
Insecta	Diptera	Empididae	*Rhamphomyia*			■	
Insecta	Diptera	Ephydridae			■	■	
Insecta	Diptera	Heleomyzidae		■			■

Class	Order	Family	Genus	EE	LE	ME	TR
Insecta	Diptera	Lauxaniidae		■	■	■	
Insecta	Diptera	Mycetophilidae				■	■
Insecta	Diptera	Phoridae			■	■	
Insecta	Diptera	Pipunculidae					■
Insecta	Diptera	Sarcophagidae					■
Insecta	Diptera	Scatopsidae			■	■	■
Insecta	Diptera	Sciaridae			■	■	■
Insecta	Diptera	Sphaeroceridae		■	■	■	■
Insecta	Diptera	Syrphidae		■		■	
Insecta	Diptera	Tachinidae		■			
Insecta	Diptera	Torymidae		■			
Insecta	Hemiptera	Acanthosomatidae				■	
Insecta	Hemiptera	Adelgidae			■	■	■
Insecta	Hemiptera	Anthocoridae				■	
Insecta	Hemiptera	Aphididae		■	■	■	■
Insecta	Hemiptera	Auchenorrhyncha		■			
Insecta	Hemiptera	Burprestidae		■			
Insecta	Hemiptera	Cercopidae		■	■	■	■
Insecta	Hemiptera	Cicadellidae		■	■	■	■
Insecta	Hemiptera	Coccoidea		■		■	
Insecta	Hemiptera	Delphacidae		■			
Insecta	Hemiptera	Fulgoroidea		■	■		
Insecta	Hemiptera	Lygaeidae		■	■		
Insecta	Hemiptera	Membracidae					■
Insecta	Hemiptera	Miridae		■	■	■	■
Insecta	Hemiptera	Pentatomidae			■	■	■
Insecta	Hemiptera	Psyllidae		■	■	■	■
Insecta	Hemiptera	Saldidae		■			
Insecta	Hemiptera	Sternorrhyncha		■			
Insecta	Hemiptera	Tingidae			■	■	■
Insecta	Heteroptera			■			
Insecta	Hymenoptera	Aphelenidae				■	
Insecta	Hymenoptera	Apidae		■	■		
Insecta	Hymenoptera	Bethylidae			■		■
Insecta	Hymenoptera	Braconidae		■	■	■	■
Insecta	Hymenoptera	Ceraphronidae		■		■	
Insecta	Hymenoptera	Chalcidoidea		■	■	■	■
Insecta	Hymenoptera	Cynipidae		■		■	■
Insecta	Hymenoptera	Diapriidae		■	■	■	■
Insecta	Hymenoptera	Dryinidae		■			
Insecta	Hymenoptera	Encyrtidae		■		■	
Insecta	Hymenoptera	Eulophidae		■		■	■
Insecta	Hymenoptera	Figitidae		■			
Insecta	Hymenoptera	Formicidae		■	■	■	■

**Class**	**Order**	**Family**	**Genus**	**EE**	**LE**	**ME**	**TR**
Insecta	Hymenoptera	Ichneumonidae		■	■	■	■
Insecta	Hymenoptera	Megaspilidae		■	■	■	■
Insecta	Hymenoptera	Mymaridae		■	■	■	
Insecta	Hymenoptera	Platygastridae		■	■		■
Insecta	Hymenoptera	Proctotrupidae		■		■	■
Insecta	Hymenoptera	Pteromalidae		■	■	■	
Insecta	Hymenoptera	Scelionidae		■		■	■
Insecta	Hymenoptera	Sphecidae		■			
Insecta	Hymenoptera	Symphyta			■		
Insecta	Hymenoptera	Tenthredinidae		■	■		
Insecta	Hymenoptera	Trichogrammatidae		■			
Insecta	Hymenoptera	Vespidae				■	
Insecta	Lepidoptera	Arctiidae				■	
Insecta	Lepidoptera	Coleophoridae				■	
Insecta	Lepidoptera	Crambidae			■	■	■
Insecta	Lepidoptera	Geometridae		■	■	■	■
Insecta	Lepidoptera	Lasiocampidae		■			
Insecta	Lepidoptera	Lymantriidae			■	■	■
Insecta	Lepidoptera	Noctuidae				■	
Insecta	Lepidoptera	Nymphalidae				■	
Insecta	Lepidoptera	Tineidae				■	
Insecta	Neuroptera	Tortricidae		■			
Insecta	Neuroptera	Chrysopidae/Hemerobiidae		■	■	■	■
Insecta	Neuroptera	Coniopterygidae				■	
Insecta	Neuroptera	Coniopterygidae	*Semidalis*				■
Insecta	Neuroptera	Hemerobiidae					■
Insecta	Odonata	Coenagrionidae	*Ishnura*	■			
Insecta	Psocoptera	Caeciliusidae			■	■	
Insecta	Psocoptera	Ectopsocidae			■		■
Insecta	Psocoptera	Elipsocidae			■		■
Insecta	Psocoptera	Lachesillidae			■		
Insecta	Psocoptera	Liposcelidae				■	
Insecta	Psocoptera	Myopsocidae			■		
Insecta	Psocoptera	Psocidae			■	■	■
Insecta	Psocoptera	Psocoptera		■	■	■	■
Insecta	Psocoptera	Stenopsocidae			■		
Insecta	Raphidioptera	Raphidiidae			■		
Insecta	Thysanoptera	Phlaeothripidae		■	■	■	■
Insecta	Thysanoptera	Thripidae		■	■	■	■
Arachnida	Araneae			■	■	■	■
Arachnida	Opiliones			■		■	■
Arachnida	Pseudoscorpiones				■	■	■
Chilopoda						■	
Diplopoda				■		■	■

Class	Order	Family	Genus	EE	LE	ME	TR
Diplopoda	Spirobolida	Spirobolida					
Entognatha	Collembola	Entomobryidae					
Entognatha	Collembola	Hypogastruridae					
Entognatha	Collembola	Isotomidae					
Entognatha	Collembola	Neanuridae					
Entognatha	Collembola	Onychiuridae					
Entognatha	Collembola	Sminthuridae					
Entognatha	Collembola	Tomoceridae					
Gastropoda							
