# Supplementary material for: Shifting food web structure during dam removal—Disturbance and recovery during a major restoration action
Source: PLoS One. 2020 Sep 29;15(9):e0239198. doi: 10.1371/journal.pone.0239198 (PMC7523948; doi:10.1371/journal.pone.0239198)
Supplement: S3 Table — (PDF) [file pone.0239198.s007.pdf]

S3 Table. Body measurement to dry mass (DM) conversions for all encountered salmonid prey taxa. Constants a and b provided for the power equation DM = a*X^b. All constants were converted if they were reported in log form in the original source. Stage refers to life stage: A = adult, L = larva, N = nymph, I = immature, F = fry, P = pupa, and U = unknown. X refers to body measurement used for conversion (BL = body length, HW = head capsule width) and is reported in mm. Surrogate refers to the taxa/life-stage equation substituted if one could not be found for the specified taxa. Ref refers to the source reference for the equation, with full citations given below the table. If multiple species regressions were given within a single source (but none at the appropriate level), regression lines were averaged. If there is no reference number, a new equation was developed by the authors based on their own data.

Order	Family	Stage	X	Surrogate	a	b	Ref	Notes
**Phylum: Annelida**								
**Class: Clitellata**								
Branchiobdellida		U	BL	Hirudinea	0.019	2.108	26	
**Class: Hirudinea**		U	BL		0.019	2.108	26	
**Class: Oligochaeta**		U	BL		0.0016	2.681	6	Equation transformed from graph; terrestrial specimens
**Phylum: Arthropoda**								
**Class: Acari**		A/N	BL		0.0397	2.761	17	Equation converted to mg; terrestrial specimens
		L	BL	Acari - A/N	0.0397	2.761	17	
		U	BL	Acari - A/N	0.0397	2.761	17	
Oribatida		A	BL		0.0516	2.79	17	Equation converted to mg; terrestrial specimens
Trombidiformes	Arrenuridae	A	BL	Acari - A/N	0.0397	2.761	17	
Trombidiformes	Hydrovolziidae	A	BL	Acari - A/N	0.0397	2.761	17	
Trombidiformes	Hydryphantidae	A	BL	Acari - A/N	0.0397	2.761	17	
Trombidiformes	Hydryphantidae	L	BL	Acari - A/N	0.0397	2.761	17	
Trombidiformes	Hygrobatidae	A	BL	Acari - A/N	0.0397	2.761	17	
Trombidiformes	Hygrobatidae	L	BL	Acari - A/N	0.0397	2.761	17	
Trombidiformes	Lebertiidae	A	BL	Acari - A/N	0.0397	2.761	17	
Trombidiformes	Limnocharidae	A	BL	Acari - A/N	0.0397	2.761	17	
Trombidiformes	Mideopsidae	A	BL	Acari - A/N	0.0397	2.761	17	
Trombidiformes	Oxidae	A	BL	Acari - A/N	0.0397	2.761	17	
Trombidiformes	Pionidae	A	BL	Acari - A/N	0.0397	2.761	17	
Trombidiformes	Protziidae	A	BL	Acari - A/N	0.0397	2.761	17	
Trombidiformes	Sperchonidae	A	BL	Acari - A/N	0.0397	2.761	17	
Trombidiformes	Sperchonidae	L	BL	Acari - A/N	0.0397	2.761	17	
Trombidiformes	Torrenticolidae	A	BL	Acari - A/N	0.0397	2.761	17	
Trombidiformes	Unionicolidae	A	BL	Acari - A/N	0.0397	2.761	17	
**Class: Arachnida**								

Order	Family	Stage	X	Surrogate	a	b	Ref	Notes
Acarina		U	BL		0.0397	2.761	17	
Araneae		U	BL		0.0433	2.532	8	
Opiliones		U	BL		0.0405	2.916	9	
Pseudoscorpiones		U	BL		0.057	2.589	11	From neotropical specimens
**Class: Chilopoda**		I	BL	Chilopoda - U	0.0036	2.626	9	
		U	BL		0.0036	2.626	9	
Geophilomorpha		U	BL	Chilopoda - U	0.0036	2.626	9	
Geophilomorpha		U	BL	Chilopoda - U	0.0036	2.626	9	
Lithobiomorpha		U	BL	Chilopoda - U	0.0036	2.626	9	
**Class: Crustacea**								
Cladocera		U	BL		0.0058	2.653	3	
Copepoda		U	BL		0.007	2.399	3	
Copepoda	Caligidae	U	BL	Copepoda - U	0.007	2.399	3	
Decapoda		U	BL		0.0147	3.626	1	BL = carapace length
Decapoda	Astacidae	U	BL	Decapoda - U	0.0147	3.626	1	
Isopoda		U	HW		0.666	3.292	10	
Isopoda	Asellidae	U	HW	Isopoda - U	0.666	3.292	10	
Ostracoda		U	BL	Cladocera - U	0.0058	2.653	3	
**Class: Diplopoda**		I	BL	Diplopoda - U	0.0001	3.909	9	
		U	BL		0.0001	3.909	9	
Chordeumatida	Chordeumatida	U	BL	Diplopoda - U	0.0001	3.909	9	
Polydesmida		U	BL	Diplopoda - U	0.0001	3.909	9	
Polyzoniida		U	BL	Diplopoda - U	0.0001	3.909	9	
Spirobolida	Spirobolida	U	BL	Diplopoda - U	0.0001	3.909	9	
**Class: Entognatha**								
Collembola		U	BL		0.0056	2.809	8	
Collembola	Entomobryidae	U	BL	Collembola - U	0.0056	2.809	8	
Collembola	Entomobryidae	U	BL	Collembola - U	0.0024	3.676	9	
Collembola	Hypogastruridae	U	BL	Collembola - U	0.0056	2.809	8	
Collembola	Isotomidae	A	BL	Collembola - U	0.0024	3.676	9	
Collembola	Isotomidae	U	BL	Collembola - U	0.0056	2.809	8	
Collembola	Onychiuridae	U	BL	Collembola - U	0.0056	2.809	8	
Collembola	Poduridae	A	BL	Collembola - U	0.0024	3.676	9	

Order	Family	Stage	X	Surrogate	a	b	Ref	Notes
Collembola	Poduridae	U	BL	Collembola - U	0.0056	2.809	8	
Collembola	Sminthuridae	A	BL		0.0024	3.676	9	
Collembola	Sminthuridae	U	BL	Collembola - U	0.0056	2.809	8	
Collembola	Tomoceridae	U	BL	Collembola - U	0.0056	2.809	8	
**Class: Insecta**								
Archaeognatha		N	BL	Archaeognatha - N	0.004169	3.188	25	From widely varying nymph growth stages (pers. comm)
Archaeognatha	Machilidae	A	BL	Archaeognatha - N	0.004169	3.188	25	
Archaeognatha	Machilidae	N	BL	Archaeognatha - N	0.004169	3.188	25	
Coleoptera		A	HW		1.9196	2.852		New equation R^2=0.99; aquatic and terrestrial specimens
Coleoptera		L	BL		0.0077	2.91	1	Mix of aquatic and terrestrial specimens
Coleoptera		L	HW		3.5956	2.738		New equations R^2=0.99
Coleoptera	"Other"	A	HW		2.014	2.891	10	
Coleoptera	Anobiidae	A	BL	Dyticidae - A	0.0618	2.502	1	
Coleoptera	Anobiidae	A	HW	Coleoptera "other" - A	2.014	2.891	10	
Coleoptera	Bostrichidae	A	HW	Coleoptera "other" - A	2.014	2.891	10	
Coleoptera	Cantharidae	A	BL	Cantheroidea - A	0.007627	2.935	19	
Coleoptera	Cantharidae	A	HW	Coleoptera "other" - A	2.014	2.891	10	
Coleoptera	Cantharidae	L	BL	Carabidae - L	0.0338	2.162	9	
Coleoptera	Cantharidae	L	HW	Carabidae - L	2.591	2.341	10	
Coleoptera	Carabidae	A	HW		1.258	3.56	10	
Coleoptera	Carabidae	L	HW		2.591	2.341	10	
Coleoptera	Cerambycidae	A	HW		2.686	2.941	10	
Coleoptera	Cerambycidae	L	HW	Carabidae - L	2.591	2.341	10	
Coleoptera	Cerylonidae	A	HW	Coleoptera "other" - A	2.014	2.891	10	
Coleoptera	Chrysomelidae	A	HW		1.343	3.27	10	
Coleoptera	Chrysomelidae	L	HW		14.981	3.67	1	Based on *Galerucella nymphaeae*
Coleoptera	Clambidae	A	HW	Coleoptera "other" - A	2.014	2.891	10	
Coleoptera	Coccinellidae	A	HW	Coleoptera "other" - A	2.014	2.891	10	
Coleoptera	Coccinellidae	L	HW	Carabidae - L	2.591	2.341	10	
Coleoptera	Cryptophagidae	A	HW	Coleoptera "other" - A	2.014	2.891	10	
Coleoptera	Curculionidae	A	BL		0.1281	2.254	9	
Coleoptera	Curculionidae	A	HW	Coleoptera "other" - A	2.014	2.891	10	
Coleoptera	Dytiscidae	A	BL		0.0618	2.502	15	

Order	Family	Stage	X	Surrogate	a	b	Ref	Notes
Coleoptera	Dytiscidae	A	HW	Coleoptera "other" - A	2.014	2.891	10	
Coleoptera	Dytiscidae	L	BL		0.0157	3.03	1	
Coleoptera	Dytiscidae	L	HW	Elmidae - L	3.1398	2.488	1	
Coleoptera	Elateridae	A	HW	Coleoptera "other" - A	2.014	2.891	10	
Coleoptera	Elateridae	L	HW	Elmidae - L	3.1398	2.488	1	
Coleoptera	Elmidae	A	BL		0.00093	6.18	15	Based on *Elmis* spp.
Coleoptera	Elmidae	A	HW	Coleoptera "other" - A	2.014	2.891	10	
Coleoptera	Elmidae	L	BL		0.0074	2.879	1	
Coleoptera	Elmidae	L	HW		3.1398	2.488	1	Average regression across all species
Coleoptera	Erotylidae	A	HW	Coleoptera "other" - A	2.014	2.891	10	
Coleoptera	Gyrinidae	A	BL	Dyticidae - A	0.0618	2.502	1	
Coleoptera	Gyrinidae	A	HW	Coleoptera "other" - A	2.014	2.891	10	
Coleoptera	Haliplidae	A	BL		0.0271	2.744	23	
Coleoptera	Haliplidae	L	BL	Coleoptera - L	0.0077	2.91	1	
Coleoptera	Hydraenidae	A	HW	Coleoptera "other" - A	2.014	2.891	10	
Coleoptera	Hydrophilidae	A	HW	Coleoptera "other" - A	2.014	2.891	10	
Coleoptera	Hydrophilidae	L	HW	Elmidae - L	3.1398	2.488	1	
Coleoptera	Latridiidae	A	BL	Coleoptera "other" - A	0.0664	2.192	9	
Coleoptera	Latridiidae	A	HW	Coleoptera "other" - A	2.014	2.891	10	
Coleoptera	Latridiidae	L	BL	Elmidae - L	0.0074	2.879	1	
Coleoptera	Leiodidae	A	HW	Coleoptera "other" - A	2.014	2.891	10	
Coleoptera	Melandryidae	A	HW	Coleoptera "other" - A	2.014	2.891	10	
Coleoptera	Melyridae	A	HW	Coleoptera "other" - A	2.014	2.891	10	
Coleoptera	Mordellidae	A	HW	Coleoptera "other" - A	2.014	2.891	10	
Coleoptera	Nitidulidae	A	HW	Coleoptera "other" - A	2.014	2.891	10	
Coleoptera	Ptiliidae	A	HW	Coleoptera "other" - A	2.014	2.891	10	
Coleoptera	Salpingidae	A	HW	Coleoptera "other" - A	2.014	2.891	10	
Coleoptera	Salpingidae	L	HW	Elmidae - L	3.1398	2.488	1	
Coleoptera	Scolytinae	A	HW	Coleoptera "other" - A	2.014	2.891	10	
Coleoptera	Scraptiidae	A	HW	Coleoptera "other" - A	2.014	2.891	10	
Coleoptera	Silvanidae	A	HW	Coleoptera "other" - A	2.014	2.891	10	
Coleoptera	Staphylinidae	A	BL		0.001	4.026	18	
Coleoptera	Staphylinidae	A	HW		1.309	2.642	10	

Order	Family	Stage	X	Surrogate	a	b	Ref	Notes
Coleoptera	Staphylinidae	L	HW	Elmidae - L	3.1398	2.488	1	
Coleoptera	Tenebrionidae	A	HW		1.102	3.631	10	
Coleoptera	Throscidae	A	BL	Coleoptera "other" - A	0.0664	2.192	9	
Coleoptera	Throscidae	A	HW	Coleoptera "other" - A	2.014	2.891	10	
Coleoptera	Throscidae	L	BL	Chrysomellidae - L	0.0392	3.111	1	
Coleoptera	Zopheridae	L	HW	Elmidae - L	3.1398	2.488	1	
Dermaptera		A/N	HW		0.345	5.379	10	
Dermaptera	Forficulidae	A	BL		0.0015	3.497	9	Assumes adult
Dermaptera	Forficulidae	A	HW	Dermaptera - A/N	0.345	5.379	10	
Diptera		A	HW		1.3775	2.271		New equation R^2=0.82, aquatic and terrestrial specimens
Diptera	Sub-order: Brachycera	A	HW		0.655	2.526	10	Terrestrial specimens
Diptera	Sub-order: Nematocera	A	HW		3.942	3.106	10	Terrestrial specimens
Diptera		L	BL		0.0025	2.692	1	Mix of aquatic and terrestrial specimens
Diptera		L	HW		1.0388	2.285		New equation R^2=0.81
Diptera		P	HW	Diptera - L	1.0388	2.285		Used same constants as for larvae
Diptera	Anisopodidae	A	HW	Nematocera - A	3.942	3.106	10	
Diptera	Anthomyiidae	A	HW	Brachycera - A	0.655	2.526	10	
Diptera	Asilidae	A	HW	Brachycera - A	0.655	2.526	10	
Diptera	Athericidae	L	HW	Empididae - L	0.989	2.602	1	
Diptera	Bibionidae	A	HW	Nematocera - A	3.942	3.106	10	
Diptera	Blephariceridae	L	HW		0.1761	2.98	21	
Diptera	Cecidomyiidae	A	HW	Nematocera - A	3.942	3.106	10	
Diptera	Cecidomyiidae	L	HW	Blephariceridae - L	0.1761	2.98	1	
Diptera	Ceratopogonidae	A	BL	Nematocera - A	0.1	1.57	18	
Diptera	Ceratopogonidae	A	HW	Nematocera - A	3.942	3.106	10	
Diptera	Ceratopogonidae	L	BL		0.0025	2.469	1	
Diptera	Ceratopogonidae	L	HW		1.3044	2.359	1	Based on one species
Diptera	Ceratopogonidae	P	HW	Ceratopogonidae - L	1.3044	2.359	1	
Diptera	Chironomidae	A	BL		0.00215	2.71	13	Adult assumed
Diptera	Chironomidae	A	HW	Nematocera - A	3.942	3.106	10	
Diptera	Chironomidae	L	BL		0.0018	2.617	1	
Diptera	Chironomidae	L	HW		2.784	2.835	1	
Diptera	Chironomidae	P	HW		2.5	2.97	21	

Order	Family	Stage	X	Surrogate	a	b	Ref	Notes
Diptera	Culicidae	A	HW	Nematocera - A	3.942	3.106	10	
Diptera	Culicidae	L	HW	Empididae - L	0.989	2.602	1	
Diptera	Deuterophlebiidae	A	HW	Nematocera - A	3.942	3.106	10	
Diptera	Deuterophlebiidae	L	HW	Blephariceridae - L	0.1761	2.98	1	
Diptera	Deuterophlebiidae	P	HW	Blephariceridae - L	0.1761	2.98	1	
Diptera	Dixidae	A	HW	Nematocera - A	3.942	3.106	10	
Diptera	Dixidae	L	HW	Chironomidae - L	2.784	2.835	1	
Diptera	Dixidae	P	HW	Chironomidae - L	2.784	2.835	1	
Diptera	Dolichopodidae	A	BL	Brachycera - A	0.0304	2.63	9	
Diptera	Dolichopodidae	A	HW	Brachycera - A	0.655	2.526	10	
Diptera	Dolichopodidae	L	BL	Empididae - L	0.0054	2.546	1	
Diptera	Dolichopodidae	L	HW	Empididae - L	0.989	2.602	1	
Diptera	Empididae	A	BL	Brachycera - A	0.0304	2.63	9	Assumes adult
Diptera	Empididae	A	HW	Brachycera - A	0.655	2.526	10	
Diptera	Empididae	L	BL		0.0054	2.54	1	
Diptera	Empididae	L	HW		0.989	2.602	1	
Diptera	Empididae	P	HW	Empididae - L	0.989	2.602	1	
Diptera	Ephydridae	A	HW	Brachycera - A	0.655	2.526	10	
Diptera	Ephydridae	L	HW	Simulidae - L	1.3824	3.161	1	
Diptera	Ephydridae	P	HW	Simulidae - L	1.3824	3.161	1	
Diptera	Heleomyzidae	A	HW	Brachycera - A	0.655	2.526	10	
Diptera	Lauxaniidae	A	HW	Brachycera - A	0.655	2.526	10	
Diptera	Limoniidae	L	HW	Limoniidae - L	2.121	1.305	24	Based on one species
Diptera	Muscidae	A	BL	Brachycera - A	0.0304	2.63	9	
Diptera	Muscidae	A	HW	Brachycera - A	0.655	2.526	10	
Diptera	Muscidae	L	BL	Empididae - L	0.0054	2.546	1	
Diptera	Muscidae	L	HW	Empididae - L	0.989	2.602	1	
Diptera	Mycetophilidae	A	HW	Nematocera - A	3.942	3.106	10	
Diptera	Mycetophilidae	L	HW	Ceratopogonidae - L	1.3044	2.359	1	
Diptera	Pelecorhynchidae	L	HW	Chironomidae - L	2.784	2.835	1	
Diptera	Phoridae	A	HW	Brachycera - A	0.655	2.526	10	
Diptera	Phoridae	L	HW	Blephariceridae - L	0.1761	2.98	1	
Diptera	Pipunculidae	A	HW	Brachycera - A	0.655	2.526	10	

Order	Family	Stage	X	Surrogate	a	b	Ref	Notes
Diptera	Psychodidae	A	HW	Nematocera - A	3.942	3.106	10	
Diptera	Psychodidae	L	HW	Empididae - L	0.989	2.602	1	
Diptera	Psychodidae	P	HW	Empididae - L	0.989	2.602	1	
Diptera	Ptychopteridae	L	HW	Chironomidae - L	2.784	2.835	1	
Diptera	Sarcophagidae	A	HW	Brachycera - A	0.655	2.526	10	
Diptera	Sarcophagidae	L	HW	Blephariceridae - L	0.1761	2.98	1	
Diptera	Sarcophagidae	P	HW	Blephariceridae - L	0.1761	2.98	1	
Diptera	Scatopsidae	A	HW	Nematocera - A	3.942	3.106	10	
Diptera	Sciaridae	A	HW	Nematocera - A	3.942	3.106	10	
Diptera	Sciaridae	L	HW	Empididae - L	0.989	2.602	1	
Diptera	Sciomyzidae	L	HW	Empididae - L	0.989	2.602	1	
Diptera	Simuliidae	A	HW	Nematocera - A	3.942	3.106	10	
Diptera	Simuliidae	L	HW		1.3824	3.161	1	
Diptera	Simuliidae	P	HW	Simulidae - L	1.3824	3.161	1	
Diptera	Sphaeroceridae	A	BL	Brachycera - A	0.0304	2.63	9	
Diptera	Sphaeroceridae	A	HW	Brachycera - A	0.655	2.526	10	
Diptera	Sphaeroceridae	L	BL	Chironomidae - L	2.617	0.0018	1	
Diptera	Stratiomyidae	A	HW	Brachycera - A	0.655	2.526	10	
Diptera	Stratiomyidae	L	HW	Empididae - L	0.989	2.602	1	
Diptera	Syrphidae	A	HW	Brachycera - A	0.655	2.526	10	
Diptera	Syrphidae	L	HW	Empididae - L	0.989	2.602	1	
Diptera	Tachinidae	A	HW	Brachycera - A	0.655	2.526	10	
Diptera	Thaumaleidae	L	HW	Chironomidae - L	2.784	2.835	1	
Diptera	Tipulidae	A	HW	Nematocera - A	3.942	3.106	10	
Diptera	Tipulidae	L	HW	Simulidae - L	1.3824	3.161	1	
Diptera	Tipulidae	P	HW	Simulidae - L	1.3824	3.161	1	
Diptera	Trichoceridae	L	HW	Empididae - L	0.989	2.602	1	
Ephemeroptera		A	BL		0.014	2.49	18	
Ephemeroptera		L	HW		0.7492	2.84	24	
Ephemeroptera	Ameletidae	A	BL	Ephemeroptera - A	0.014	2.49	18	
Ephemeroptera	Ameletidae	L	BL		0.0077	2.588	1	
Ephemeroptera	Ameletidae	L	HW		0.962	2.9	1	
Ephemeroptera	Ameletidae	U	BL	Ameletidae - L	0.0077	2.588	1	

Order	Family	Stage	X	Surrogate	a	b	Ref	Notes
Ephemeroptera	Baetidae	A	BL	Ephemeroptera - A	0.014	2.49	18	
Ephemeroptera	Baetidae	L	BL		0.0053	2.875	1	
Ephemeroptera	Baetidae	L	HW		0.8946	3.368	1	Average regression across all species
Ephemeroptera	Baetidae	U	BL	Baetidae - L	0.0053	2.875	1	
Ephemeroptera	Ephemerellidae	A	BL	Ephemeroptera - A	0.014	2.49	18	
Ephemeroptera	Ephemerellidae	L	HW		0.6597	3.343	1	Average regression across all species
Ephemeroptera	Heptageniidae	A	BL	Ephemeroptera - A	0.014	2.49	18	
Ephemeroptera	Heptageniidae	A	BL		0.0111	2.74	13	Adult assumed
Ephemeroptera	Heptageniidae	L	BL		0.0108	2.754	1	
Ephemeroptera	Heptageniidae	L	HW		0.1489	2.887	16	
Ephemeroptera	Leptophlebiidae	A	BL	Ephemeroptera - A	0.014	2.49	18	
Ephemeroptera	Leptophlebiidae	L	HW		0.4905	3.381	1	Average regression across all species
Ephemeroptera	Siphlonuridae	L	BL		0.0027	3.446	1	
Ephemeroptera	Siphlonuridae	L	HW		0.2585	4.113	1	Average regression across all species
Hemiptera		A	BL		0.09975	2.49475	18	Averaged Order values
Hemiptera		A/N	HW		2.0596	2.6657	10	Averaged regressions from Homoptera and Heteroptera
Hemiptera		L	BL		0.0108	2.734	1	
Hemiptera	Sub-order: Homoptera	A/N	HW		0.534	2.715	10	
Hemiptera	"heavy"	A/N	HW		4.238	2.664	10	
Hemiptera	"slender"	A/N	HW		3.86	2.618	10	
Hemiptera	Acanthosomatidae	A	HW	Heteroptera "heavy" - A/N	4.238	2.664	10	
Hemiptera	Acanthosomatidae	A/N	BL	Heteroptera "heavy" - A/N	0.0399	2.802	9	
Hemiptera	Adelgidae	A	HW	Homoptera - A/N	0.534	2.715	10	
Hemiptera	Anthocoridae	A	BL	Heteroptera "heavy" - A/N	0.0399	2.802	9	
Hemiptera	Anthocoridae	A	HW	Heteroptera "heavy" - A/N	4.238	2.664	10	
Hemiptera	Anthocoridae	A/N	BL	Heteroptera "heavy" - A/N	0.0399	2.802	9	
Hemiptera	Anthocoridae	L	HW	Heteroptera "heavy" - A/N	4.238	2.664	10	
Hemiptera	Aphididae	A	HW	Homoptera - A/N	0.534	2.715	10	
Hemiptera	Aphididae	A/N	BL		0.0598	1.724	9	
Hemiptera	Aphididae	L	HW	Homoptera - A/N	0.534	2.715	10	
Hemiptera	Aphididae	U	HW	Homoptera - A/N	0.534	2.715	10	
Hemiptera	Cercopidae	A	BL	Cicadellidae - A	0.079	2.229	18	
Hemiptera	Cercopidae	A	HW	Homoptera - A/N	0.534	2.715	10	

Order	Family	Stage	X	Surrogate	a	b	Ref	Notes
Hemiptera	Cercopidae	L	HW	Homoptera - A/N	0.534	2.715	10	
Hemiptera	Cercopidae	U	BL	Cicadellidae - A	0.079	2.229	18	
Hemiptera	Cicadellidae	A	BL		0.079	2.229	18	
Hemiptera	Cicadellidae	A	HW	Homoptera - A/N	0.534	2.715	10	
Hemiptera	Cicadellidae	L	HW	Homoptera - A/N	0.534	2.715	10	
Hemiptera	Cicadellidae	U	BL	Cicadellidae - A	0.079	2.229	18	
Hemiptera	Cixiidae	A	HW	Homoptera - A/N	0.534	2.715	10	
Hemiptera	Coccoidea	A/N	BL	Homoptera - A/N	0.0548	2.354	9	Constants for non-aphid Homoptera
Hemiptera	Coccoidea	A	HW	Homoptera - A/N	0.534	2.715	10	
Hemiptera	Corixidae	A	BL	Corixidae - L	0.0031	2.904	1	
Hemiptera	Corixidae	L	BL		0.0031	2.904	1	
Hemiptera	Corixidae	L	HW	Heteroptera "slender" - A/N	3.86	2.618	10	
Hemiptera	Delphacidae	A	BL	Cicadellidae - A	0.079	2.229	18	
Hemiptera	Delphacidae	A	HW	Homoptera - A/N	0.534	2.715	10	
Hemiptera	Delphacidae	L	BL		0.0206	2.764	8	Values for all life stages
Hemiptera	Delphacidae	U	BL	Cicadellidae - A	0.079	2.229	18	
Hemiptera	Fulgoroidea	L	HW	Homoptera - A/N	0.534	2.715	10	
Hemiptera	Gerridae	L	HW	Heteroptera "slender" - A/N	3.86	2.618	10	
Hemiptera	Lygaeidae	A	HW	Heteroptera "slender" - A/N	3.86	2.618	10	
Hemiptera	Lygaeidae	A/N	BL		0.0375	2.065	8	
Hemiptera	Lygaeidae	L	HW	Heteroptera "slender" - A/N	3.86	2.618	10	
Hemiptera	Membracidae	A	HW	Homoptera - A/N	0.534	2.715	10	
Hemiptera	Miridae	A	BL		0.104664	1.491	19	
Hemiptera	Miridae	A	HW	Heteroptera "slender" - A/N	3.86	2.618	10	
Hemiptera	Miridae	L	HW	Heteroptera "slender" - A/N	3.86	2.618	10	
Hemiptera	Pentatomidae	A	HW	Heteroptera "heavy" - A/N	4.238	2.664	10	
Hemiptera	Pentatomidae	L	HW	Heteroptera "heavy" - A/N	4.238	2.664	10	
Hemiptera	Psyllidae	A	BL		0.0123	2.995	8	
Hemiptera	Psyllidae	A	HW	Homoptera - A/N	0.534	2.715	10	
Hemiptera	Psyllidae	L	BL		0.0175	2.629	8	
Hemiptera	Psyllidae	L	HW	Homoptera - A/N	0.534	2.715	10	
Hemiptera	Saldidae	A	BL		0.079	2.229	18	Assumes adult
Hemiptera	Saldidae	A	HW	Heteroptera "slender" - A/N	3.86	2.618	10	

Order	Family	Stage	X	Surrogate	a	b	Ref	Notes
Hemiptera	Saldidae	L	HW	Heteroptera "slender" - A/N	3.86	2.618	10	
Hemiptera	Thyreocoridae	A	HW	Heteroptera "heavy" - A/N	4.238	2.664	10	
Hemiptera	Thyreocoridae	L	HW	Heteroptera "heavy" - A/N	4.238	2.664	10	
Hemiptera	Tingidae	A	HW	Heteroptera "slender" - A/N	3.86	2.618	10	
Hymenoptera		A	BL		0.013787	2.696	19	
Hymenoptera		A	HW		1.999	2.09	10	Non-Formicidae
Hymenoptera		L	BL	Tenthredinidae - L	0.0113	2.816	19	
Hymenoptera	Aphelenidae	A	HW	Hymenoptera - A	1.999	2.09	10	
Hymenoptera	Apidae	A	HW	Hymenoptera - A	1.999	2.09	10	
Hymenoptera	Bethylidae	A	HW	Hymenoptera - A	1.999	2.09	10	
Hymenoptera	Braconidae	A	BL		0.021195	2.441	19	
Hymenoptera	Braconidae	A	HW	Hymenoptera - A	1.999	2.09	10	
Hymenoptera	Braconidae	L	BL	Tenthredinidae - L	0.01127	2.816	19	
Hymenoptera	Cephidae	A	HW	Hymenoptera - A	1.999	2.09	10	
Hymenoptera	Ceraphronidae	A	HW	Hymenoptera - A	1.999	2.09	10	
Hymenoptera	Chalcidoidea	A	HW	Hymenoptera - A	1.999	2.09	10	
Hymenoptera	Cynipidae	A	HW	Hymenoptera - A	1.999	2.09	10	
Hymenoptera	Diapriidae	A	HW	Hymenoptera - A	1.999	2.09	10	
Hymenoptera	Encyrtidae	A	HW	Hymenoptera - A	1.999	2.09	10	
Hymenoptera	Eulophidae	A	HW	Hymenoptera - A	1.999	2.09	10	
Hymenoptera	Figitidae	A	HW	Hymenoptera - A	1.999	2.09	10	
Hymenoptera	Formicidae	A	BL		0.027	2.666	18	
Hymenoptera	Formicidae	A	HW	Formicidae - A	0.552	2.55	10	Based on worker Formicidae
Hymenoptera	Halictidae	A	HW	Hymenoptera - A	1.999	2.09	10	
Hymenoptera	Ichneumonidae	A	BL		0.015780	2.464	19	
Hymenoptera	Ichneumonidae	A	HW	Hymenoptera - A	1.999	2.09	10	
Hymenoptera	Ichneumonidae	L	BL	Tenthredinidae - L	0.01127	2.816	19	
Hymenoptera	Megaspilidae	A	HW	Hymenoptera - A	1.999	2.09	10	
Hymenoptera	Mymaridae	A	HW	Hymenoptera - A	1.999	2.09	10	
Hymenoptera	Platygastridae	A	BL	Hymenoptera - A	0.013787	2.696	19	
Hymenoptera	Platygastridae	A	HW	Hymenoptera - A	1.999	2.09	10	
Hymenoptera	Platygastridae	L	BL	Tenthredinidae - L	0.01127	2.816	19	
Hymenoptera	Proctotrupidae	A	HW	Hymenoptera - A	1.999	2.09	10	

Order	Family	Stage	X	Surrogate	a	b	Ref	Notes
Hymenoptera	Pteromalidae	A	HW	Hymenoptera - A	1.999	2.09	10	
Hymenoptera	Scelionidae	A	HW	Hymenoptera - A	1.999	2.09	10	
Hymenoptera	Sphecidae	A	HW	Hymenoptera - A	1.999	2.09	10	
Hymenoptera	Symphyta	L	BL	Tenthredinidae - L	0.0113	2.816	19	
Hymenoptera	Tenthredinidae	A	BL	Hymenoptera - A	0.013787	2.696	19	
Hymenoptera	Tenthredinidae	A	HW	Hymenoptera - A	1.999	2.09	10	
Hymenoptera	Tenthredinidae	L	BL		0.0113	2.816	19	
Hymenoptera	Tenthredinidae	L	BL		0.01127	2.816	19	
Hymenoptera	Vespidae	A	HW	Hymenoptera - A	1.999	2.09	10	
Isoptera		A	HW		1.275	3.344	10	
Isoptera	Hodotermitidae	A	HW	Isoptera - A	1.275	3.344	10	
Lepidoptera		A	BL	Pyralidae - A	0.012	2.695	18	
Lepidoptera		A	HW		2.053	2.804	10	Based on Heterocera
Lepidoptera		L	BL	Pyralidae - L	0.0033	2.918	1	
Lepidoptera		L	HW		5.532	2.129	10	
Lepidoptera		P	HW	Lepidoptera - L	5.532	2.129	10	
Lepidoptera	Arctiidae	L	HW	Lepidoptera - L	5.532	2.129	10	
Lepidoptera	Coleophoridae	L	HW	Lepidoptera - L	5.532	2.129	10	
Lepidoptera	Crambidae	L	HW	Lepidoptera - L	5.532	2.129	10	
Lepidoptera	Geometridae	A	BL		0.015421	2.628	19	
Lepidoptera	Geometridae	A	HW	Lepidoptera - A	2.053	2.804	10	
Lepidoptera	Geometridae	L	BL		0.004115	2.625	19	
Lepidoptera	Geometridae	L	HW	Lepidoptera - L	5.532	2.129	10	
Lepidoptera	Lasiocampidae	A	HW	Lepidoptera - A	2.053	2.804	10	
Lepidoptera	Lasiocampidae	L	BL	Lepidoptera - L	0.011	2.571	9	
Lepidoptera	Lymantriidae	L	HW	Lepidoptera - L	5.532	2.129	10	
Lepidoptera	Noctuidae	A	BL		0.035543	2.499	19	
Lepidoptera	Noctuidae	A	HW	Lepidoptera - A	2.053	2.804	10	
Lepidoptera	Noctuidae	L	BL		0.004409	2.845	19	
Lepidoptera	Noctuidae	L	HW	Lepidoptera - L	5.532	2.129	10	
Lepidoptera	Notodontidae	A	HW	Lepidoptera - A	2.053	2.804	10	
Lepidoptera	Notodontidae	L	HW	Lepidoptera - L	5.532	2.129	10	
Lepidoptera	Nymphalidae	L	HW	Lepidoptera - L	5.532	2.129	10	

Order	Family	Stage	X	Surrogate	a	b	Ref	Notes
Lepidoptera	Tineidae	A	HW	Lepidoptera - A	2.053	2.804	10	
Lepidoptera	Tortricidae	A	HW	Lepidoptera - A	2.053	2.804	10	
Lepidoptera	Tortricidae	L	HW	Lepidoptera - L	5.532	2.129	10	
Megaloptera	Sialidae	A	BL	Neuroptera - A	0.007	2.739	8	
Megaloptera	Sialidae	L	BL		0.0037	2.753	1	
Megaloptera	Sialidae	L	HW		0.5137	3.022	1	Average regression based on two species
Neuroptera		A	BL		0.007	2.739	8	
Neuroptera		L	BL		0.0814	1.53	9	
Neuroptera		L	HW		0.773	2.829	10	
Neuroptera		U	HW	Neuroptera - L	0.773	2.829	10	
Neuroptera	Chrysopidae	A	BL		0.007	2.739	8	
Neuroptera	Chrysopidae	L	BL	Neuroptera - A	0.0814	1.53	9	
Neuroptera	Chrysopidae	L	HW	Neuroptera - L	0.773	2.829	10	
Neuroptera	Chrysopidae/Hemerobiidae	L	HW	Neuroptera - L	0.773	2.829	10	
Neuroptera	Coniopterygidae	A	BL	Neuroptera - A	0.007	2.739	8	
Neuroptera	Coniopterygidae	L	HW	Neuroptera - L	0.773	2.829	10	
Neuroptera	Hemerobiidae	A	BL		0.007	2.739	8	Adult assumed
Neuroptera	Hemerobiidae	L	BL	Neuroptera - L	0.0814	1.53	9	
Neuroptera	Hemerobiidae	U	HW	Neuroptera - L	0.773	2.829	10	
Odonata	Aeshnidae	L	BL		0.0082	2.813	1	
Odonata	Coenagrionidae	L	BL	Ischnura - L	0.0015	2.904	1	
Odonata	Libellulidae	L	BL	Ischnura - L	0.0015	2.904	1	
Orthoptera		A	HW	Homoptera - A	0.534	2.715	10	
Orthoptera	Acrididae	A	HW	Homoptera - A	0.534	2.715	10	
Orthoptera	Orthoptera	A	HW	Homoptera - A	0.534	2.715	10	
Orthoptera	Orthoptera	L	HW	Homoptera - A	0.534	2.715	10	
Orthoptera	Rhaphidophoridae	A	HW	Homoptera - A	0.534	2.715	10	
Plecoptera		A	BL		0.26	1.69	18	
Plecoptera		L	HW		0.9062	2.854	24	
Plecoptera	Capniidae	A	BL	Plecoptera - A	0.26	1.69	18	
Plecoptera	Capniidae	L	BL		0.0049	2.562	1	
Plecoptera	Capniidae	L	HW		0.549	2.996	1	Average regression based on all species
Plecoptera	Chloroperlidae	A	BL	Plecoptera - A	0.26	1.69	18	

Order	Family	Stage	X	Surrogate	a	b	Ref	Notes
Plecoptera	Chloroperlidae	A	BL		0.005	2.732	18	
Plecoptera	Chloroperlidae	L	BL		0.0065	2.724	1	
Plecoptera	Chloroperlidae	L	HW		0.715	2.975	16	
Plecoptera	Leuctridae	A	BL	Plecoptera - A	0.26	1.69	18	
Plecoptera	Leuctridae	L	HW		0.85	3.201	1	Based on *Leuctra* spp.
Plecoptera	Nemouridae	A	BL	Plecoptera - A	0.26	1.69	18	
Plecoptera	Nemouridae	L	HW		0.7544	2.948	1	Average regression based on all species
Plecoptera	Peltoperlidae	L	HW		0.513	3.875	1	Average regression based on all species
Plecoptera	Perlidae	A	BL	Plecoptera - A	0.26	1.69	18	
Plecoptera	Perlidae	L	HW		0.3061	3.082	1	Average regression based on all species
Plecoptera	Perlodidae	A	BL	Plecoptera - A	0.26	1.69	18	
Plecoptera	Perlodidae	L	BL		0.0196	2.742	1	
Plecoptera	Perlodidae	L	HW		0.6465	2.894	1	Average regression based on all species
Plecoptera	Perlodidae/Perlidae	L	HW		0.4219	3.091	1	Average regression of Perlodidae and Perlidae
Plecoptera	Pteronarcyidae	L	HW	Nemouridae - L	0.7544	2.948	1	
Plecoptera	Taeniopterygidae	L	HW	Nemouridae - L	0.7544	2.948	1	
Psocoptera		A	BL		0.0136	3.115	8	
Psocoptera		L	BL	Psocoptera - A	0.0136	3.115	8	
Psocoptera		P	BL	Psocoptera - A	0.0136	3.115	8	
Psocoptera	Caeciliusidae	A	BL	Psocoptera - A	0.0136	3.115	8	
Psocoptera	Ectopsocidae	A	BL	Psocoptera - A	0.0136	3.115	8	
Psocoptera	Elipsocidae	U	BL	Psocoptera - A	0.0136	3.115	8	
Psocoptera	Lachesillidae	A	BL	Psocoptera - A	0.0136	3.115	8	
Psocoptera	Liposcelidae	U	BL	Psocoptera - A	0.0136	3.115	8	
Psocoptera	Myopsocidae	A	BL	Psocoptera - A	0.0136	3.115	8	
Psocoptera	Psocidae	A	BL	Psocoptera - A	0.0136	3.115	8	
Psocoptera	Stenopsocidae	A	BL	Psocoptera - A	0.0136	3.115	8	
Raphidioptera	Raphidiidae	A	BL	Neuroptera - A	0.007	2.739	8	
Thysanoptera		A	BL		0.0071	2.537	9	
Thysanoptera		L	BL	Thysanoptera - A	0.0071	2.537	9	
Thysanoptera	Phlaeothripidae	A	BL	Thysanoptera - A	0.0071	2.537	9	
Thysanoptera	Phlaeothripidae	A	BL		0.0071	2.537	9	Assumes adult
Thysanoptera	Thripidae	A	BL	Thysanoptera - A	0.0071	2.537	9	

Order	Family	Stage	X	Surrogate	a	b	Ref	Notes
Thysanoptera	Thripidae	A	BL	Thysanoptera - A	0.0071	2.537	9	
Trichoptera		A	BL		0.01	2.9	18	
Trichoptera		L	BL		0.0056	2.839	1	
Trichoptera		L	HW		1.4346	2.52	24	
Trichoptera		P	HW		1.63	2.78	21	
Trichoptera	Apataniidae	A	BL	Trichoptera - A	0.01	2.9	18	
Trichoptera	Apataniidae	L	HW	Limnephilidae - L	2.2075	3.48	1	
Trichoptera	Brachycentridae	A	BL	Trichoptera - A	0.01	2.9	18	
Trichoptera	Brachycentridae	L	HW		2.221	3.349	1	
Trichoptera	Brachycentridae	P	HW	Trichoptera - P	1.63	2.78	21	
Trichoptera	Glossosomatidae	A	BL	Trichoptera - A	0.01	2.9	18	
Trichoptera	Glossosomatidae	L	BL		0.0082	2.958	1	
Trichoptera	Glossosomatidae	L	HW		2.609	2.979	16	Based on *Glossosoma* spp.
Trichoptera	Glossosomatidae	P	HW	Trichoptera - P	1.63	2.78	21	
Trichoptera	Goeridae	L	HW		2.3662	3.576	16	
Trichoptera	Hydropsychidae	A	BL	Trichoptera - A	0.01	2.9	18	
Trichoptera	Hydropsychidae	L	BL		0.0046	2.926	1	
Trichoptera	Hydropsychidae	L	HW		1.2542	2.824	1	Average regression based on all species
Trichoptera	Hydropsychidae	P	HW	Trichoptera - P	1.63	2.78	21	
Trichoptera	Hydroptilidae	A	BL	Trichoptera - A	0.01	2.9	18	
Trichoptera	Hydroptilidae	A	BL	Trichoptera - A	0.01	2.9	18	
Trichoptera	Hydroptilidae	L	BL	Rhyacophilidae - L	0.0099	2.48	1	
Trichoptera	Hydroptilidae	L	HW	Rhyacophilidae - L	3.4497	3.823	1	
Trichoptera	Hydroptilidae	P	HW	Trichoptera - P	1.63	2.78	21	
Trichoptera	Lepidostomatidae	A	BL	Trichoptera - A	0.01	2.9	18	
Trichoptera	Lepidostomatidae	L	HW		1.666	2.987	1	
Trichoptera	Lepidostomatidae	P	HW	Trichoptera - P	1.63	2.78	21	
Trichoptera	Leptoceridae	L	HW		2.2497	3.297	1	Average regression based on all species
Trichoptera	Limnephilidae	A	BL	Trichoptera - A	0.01	2.9	18	
Trichoptera	Limnephilidae	L	BL		0.004	2.933	1	
Trichoptera	Limnephilidae	L	HW		2.2075	3.48	1	Average regression based on all species
Trichoptera	Limnephilidae	P	HW	Trichoptera - P	1.63	2.78	21	
Trichoptera	Philopotamidae	A	BL	Trichoptera - A	0.01	2.9	18	

Order	Family	Stage	X	Surrogate	a	b	Ref	Notes
Trichoptera	Philopotamidae	L	HW		1.2729	3.115	1	Average regression based on all species
Trichoptera	Philopotamidae	P	HW	Trichoptera - P	1.63	2.78	21	
Trichoptera	Polycentropodidae	L	HW		1.0365	3.941	1	Average regression based on all species
Trichoptera	Rhyacophilidae	A	BL	Trichoptera - A	0.01	2.9	18	
Trichoptera	Rhyacophilidae	L	BL		0.0099	2.48	1	
Trichoptera	Rhyacophilidae	L	HW		3.4497	3.823	1	Average regression based on all species
Trichoptera	Rhyacophilidae	P	HW	Trichoptera - P	1.63	2.78	21	
Trichoptera	Uenoidae	L	HW	Limnephilidae - L	2.2075	3.48	1	
**Class: Malacostraca**								
Amphipoda		I	BL	Amphipoda - U	0.0058	3.015	1	
Amphipoda		U	BL		0.0058	3.015	1	
Amphipoda	Corophiidae	U	BL		0.0023	3.164	2	
Amphipoda	Crangonyctidae	U	BL		0.0034	3.005	1	Average regression based on all species
Amphipoda	Gammaridae	U	BL		0.0077	2.871	1	Average regression based on all species
Amphipoda	Gammaridae	U	BL		0.0049	3.001	1	
Amphipoda	Hyalellidae	U	BL	Gammaridae - U	0.0077	2.871	1	
Isopoda	Sphaeromatidae	U	BL	Isopoda - U	0.0101	2.844	9	
Mysida		U	BL		0.000176	3.02	22	
**Phylum: Chordata**								
		Egg	BL	Salmonidae - egg	0.3643	2.755	7	Original equation calculated wet weight; used pDM (0.414) to convert to DW; based on coho/chum eggs
		U	BL	Salmonidae - I	0.00003	2.778	21	
**Class: Actinopterygii**								
Gasterosteiformes	Gasterosteidae	U	BL		0.0086	3.04	14	
Salmoniformes	Salmonidae	I	BL		0.00003	2.778	21	
Scorpaeniformes	Cottidae	A	BL		0.002	3.04	21	
Scorpaeniformes	Cottidae	I	BL		0.002	3.04	21	
Scorpaeniformes	Cottidae	F	BL		0.0037	3.3	14	
**Class: Amphibia**								
Anura		I	BL		0.0069	3.231	5	Using snout-vent length, equation given in wet weight, used assumed pDM (0.095) to convert to DW; based on *Hyla* spp, *Pseudacris* spp, and *Rana* spp
Anura	Leiopelmatidae	L	BL	Anura - Is	0.0069	3.231	5	
**Class: Cephalaspidomorphi**								

Order	Family	Stage	X	Surrogate	a	b	Ref	Notes
Petromyzontiformes	Petromyzontidae	L	BL		0.0015	2.655	20	Equation given in wet weight, used assumed pDM (0.217) to convert to DW
**Phylum: Mollusca**								
**Class: Bivalvia**								
Veneroida	Sphaeriidae	L	BL		0.0163	2.477	1	
**Class: Gastropoda**								
Basommatophora		U	BL		0.000023	2.7567	4	Calculated average from found taxa
Basommatophora	Ancylidae	U	BL	Physidae - all	0.000009	3.07	4	
Basommatophora	Lymnaeidae	All	BL		0.00003	2.67	4	Based on *Lymnaea palustris*; shell-free dry weight
Basommatophora	Lymnaeidae	U	BL		0.00003	2.67	4	
Basommatophora	Physidae	All	BL		0.000009	3.07	4	Based on *Physa fontinalis*; shell-free dry weight
Basommatophora	Physidae	U	BL		0.000009	3.07	4	
Basommatophora	Planorbidae	All	BL		0.00003	2.53	4	Based on *Anisus rotundatus*; shell-free dry weight
Basommatophora	Planorbidae	U	BL		0.00003	2.53	4	
Gastropoda		I	BL		0.0124	2.842	21	From slugs, ranging in 16-60 mm length
Gastropoda		U	BL	Gastropoda - I	0.0124	2.842	21	
Heterostropha	Valvatidae	U	BL	Planorbidae - all	0.00003	2.53	4	
Neotaenioglossa	Hydrobiidae	U	BL		0.1229	2.451	24	Based on *Potamopyrgus*, includes shell weight; BL = shell height.
Stylommatophora	Arionidae	U	BL	Gastropoda - I	0.0124	2.842	21	
Stylommatophora	Limacidae	U	BL	Gastropoda - I	0.0124	2.842	21	
Stylommatophora	Succineidae	U	BL	Gastropoda - I	0.0124	2.842	21	
**Phylum: Nematoda**								
		U	BL		0.0009	0.4	12	Equation reported backwards (Y=BL, X=DW), converted coefficients so that DW was the responding variable
**Phylum: Nematomorpha**								
		U	BL	Nematoda - U	0.0009	0.4	12	
**Phylum: Platyhelminthes**								
**Class: Turbellaria**								
Tricladida	Planariidae	U	BL	Turbellaria - U	0.0082	2.168	1	
Turbellaria		U	BL		0.0082	2.168	1	

References

1. Benke, A.C., Huryn, A.D., Smock, L.A., and Wallace, J.B. 1999. Length-Mass relationships for freshwater macroinvertabrates in North America with particular reference to southeastern United States. Journal of the North American Benthological Society 18(3):308-344
2. Boates, J.S., and Smith, P.C. 1979. Length-weight relationships, energy content and the efforts of predation on *Corophium volutator* (Pallas)(Crustacea: amphipoda). Proceedings of the Nova Scotian Institute of Science 29:489-499.
3. Bottrell, H.H., Duncan, A., Gliwicz, Z.M., Herzig, G.E., Hillbricht, A., Ilkowska, H., Larrson, A., and Weglenska, T. 1976. A review of some problems in zooplankton production studies. Norwegian Journal of Zoology 24:419–456.
4. Caquet, T. 1993. Comparative life-cycle, biomass and secondary production of three sympatric freshwater gastropod species. Journal of Molluscan Studies 59:43-50.
5. Chris Davis, Personal Comm. U. Pittsburg, Pymatuning Laboratory of Ecology; data from U. Michigan ES George Reserve
6. Collins, P. T. 1992. Length-biomass relationships for terrestrial Gastropoda and Oligochaeta. American Midland Naturalist 128:404-406.
7. Fleming, I. A., and Ng, S. 1987. Evaluation of techniques for fixing, preserving, and measuring salmon eggs. Canadian Journal of Fisheries and Aquatic Sciences 44:1957-1962.
8. Gruner, D.S. 2003. Regression of length and width to predict arthropod biomass in the Hawaiian Islands. Pacific Science 57:325-336
9. Hodar, J.A. 1996. The use of regression equations for estimation of arthropod biomass in ecological studies. Acta Ecologica, 17:421-433.
10. Hodar, J.A. 1997. The use of regression equations for estimation of prey length and biomass in diet studies of insectivore vertebrates. Misc. Zool. 20(2):1-10
11. Höfer, H., and Ott, R. 2009. Estimating biomass of Neotropical spiders and other arachnids (Araneae, Opiliones, Pseudoscorpiones, Ricinulei) by mass-length regressions. Journal of Arachnology, 37:160-169.
12. Huhta, V., and Koskenniemi, A. 1975. Numbers, biomass and community respiration of soil invertebrates in spruce forests at two latitudes in Finland. Annales Zoologici Fennici 12:164-182.
13. Johnston, T.A. and Cunjak, R.A. 1999. Dry mass–length relationships for benthic insects: a review with new data from Catamaran Brook, New Brunswick, Canada. Freshwater Biology 41:653-674.
14. Kimmerer, W., Avent, S.R., Bollens, S.M., Feyrer, F., Grimaldo, L.F., Moyle, P.B., Nobriga, M. and Visintainer, T. 2005. Variability in length–weight relationships used to estimate biomass of estuarine fish from survey data. Transactions of the American Fisheries Society 134:481-495.
15. Lecerf, A. and Richardson, J.S. 2011. Assessing the functional importance of large-bodied invertebrates in experimental headwater streams. Oikos 120:950-960.
16. Meyer, E. 1989.The relationship between body length parameters and dry mass in running water invertebrates. Archiv fur Hydrobiologie 117:191-203.
17. Rogers, L. E, Buschbom, R. L., and Watson, C. R. 1976. Length-weight relationships of shrub-steppe invertebrates. Annals of the Entomological Society of America 70:51-53.
18. Sabo, J. L., Bastow, J. L., and Power, M. E. 2002. Length–mass relationships for adult aquatic and terrestrial invertebrates in a California watershed. Journal of the North American Benthological Society 21:336-343.
19. Sample, B. E., Cooper, R. J., Greer, R. D., and Whitmore, R. C. 1993. Estimation of insect biomass by length and width. American Midland Naturalist 129:234-240.
20. Schneider, J.C., Laarman, P.W., and Gowing, H. 2000. Length-weight relationships. Chapter 17 in Schneider, J.C. (ed.), Manual of Fisheries Survey Methods II: With Periodic Updates. Michigan Department of Natural Resources, Fisheries Special Report 25, Ann Arbor.
21. Sean Naman, University of British Columbia, Vancouver, B.C., unpublished data from the Cedar River, WA.
22. Sell, D.W., 1982. Size-frequency estimates of secondary production by *Mysis relicta* in Lakes Michigan and Huron. Hydrobiologia 93:69-78.
23. Smock, L.A. 1980. Relationships between body size and biomass of aquatic insects. Freshwater Biology 10: 375-383.
24. Towers, D.J., Henderson, I.M., and Veltman, C.J. 1994. Predicting dry weight of New Zealand aquatic macroinvertebrates from linear dimensions. New Zealand Journal of Marine and Freshwater Research 28: 159-166.
25. Townsend, J. M., Rimmer, C. C., Mcfarland, K. P., and Goetz, J. E. 2012. Site-specific variation in food resources, sex ratios, and body condition of an overwintering migrant songbird. The Auk 129: 683-690.
26. Ward, M. C., Willis, D. W., Herwig, B. R., Chipps, S. R., Parsons, B. G., Reed, J. R., and Hanson, M. A. 2008. Consumption estimates of walleye stocked as fry to suppress fathead minnow populations in west-central Minnesota wetlands. Ecology of Freshwater fish 17:59-70.
