# Supplementary material for: Shifting food web structure during dam removal—Disturbance and recovery during a major restoration action
Source: PLoS One. 2020 Sep 29;15(9):e0239198. doi: 10.1371/journal.pone.0239198 (PMC7523948; doi:10.1371/journal.pone.0239198)
Supplement: S4 Table — (PDF) [file pone.0239198.s008.pdf]

S4 Table. Energy density (ED) and percent dry mass (pDM) conversions for all taxa encountered in drift and fish diet samples. ED is expressed in joules per gram of dry weight. Stage refers to life stage: A = adult, L = larva, N = nymph, I = immature, F = fry, P = pupa, and U = unknown. Habitat refers to aquatic (A), Terrestrial (T) or unknown (U) origin. Surrogate refers to the taxa/life-stage equation used if one could not be found for the specified taxa. Ref refers to the source references for the equation, with full citations given below the table. If multiple species ED values were given within a single source (but none at the appropriate level), ED values were averaged, as indicated in the notes column.

Order	Stage	Habitat	Surrogate	pDM	ED	Ref	Notes
**Phylum: Annelida**							
**Class: Clitellata**							
Branchiobdellida	U	A	Hirudinea - aquatic	0.152	22845	8, 9, 25	
**Class: Hirudinea**	U	A		0.152	22845	8, 9, 25	ED values averaged across sources
**Class: Oligochaeta**	U	A		0.16	23326	8, 10	
	U	T		0.206	19117	8, 25	ED value based on Lumbricidae
**Phylum: Arthropoda**							
**Class: Acari**	A	A		0.16	27135	9	
	L	A	Acari - aquatic adult	0.16	27135	9	
	U	T		0.16	22530	18	pDM used aquatic
Oribatida	A	A	Acari - aquatic adult	0.16	27135	9	
Trombidiformes	A	A	Acari - aquatic adult	0.16	27135	9	
Trombidiformes	L	A	Acari - aquatic adult	0.16	27135	9	
**Class: Arachnida**							
Acarina	U	U	Araneae	0.308	24005	2, 24	ED values averaged across sources
Araneae	U	T		0.308	24005	2, 24	ED values averaged across sources
Opiliones	U	T		0.356	23890	2, 24	ED values averaged across sources
Pseudoscorpiones	U	T		0.155	22969	21	ED values averaged across life stages, based on single species
**Class: Chilopoda**	I	T	Chilopoda - terrestrial unknown	0.239	21852	1, 24, 25	pDM value is the average of 3 orders
	U	T		0.239	21852	1, 24, 25	ED values average of 2 sources; pDM is average of 3 orders
Geophilomorpha	U	T	Chilopoda - terrestrial unknown	0.235	21852	1, 24, 25	
Lithobiomorpha	U	T	Chilopoda - terrestrial unknown	0.201	21852	1, 24, 25	
**Class: Branchiopoda**							
Diplostraca	U	A	Cladocera and Copepoda	0.061	24121	8, 15	ED average value from source 8; pDM averaged between sources
Diplostraca	U	A	Cladocera and Copepoda	0.061	24121	8, 15	ED average value from source 8; pDM averaged between sources
**Class: Crustacea**							
Cladocera	U	A	Cladocera and Copepoda	0.061	21928	8, 15	ED average value from source 8; pDM averaged between sources
Copepoda	U	A	Cladocera and Copepoda	0.061	24121	8, 15	ED average value from source 8; pDM averaged between sources
Decapoda	U	A		0.267	16502	8	

Order	Stage	Habitat	Surrogate	pDM	ED	Ref	Notes
Isopoda	U	A		0.25	12569	8	
Ostracoda	U	A		0.349	23778	8	
**Class: Diplopoda**	I	T	Diplopoda - terrestrial unknown	0.349	15725	2, 24	
	U	T		0.349	15725	2, 24	ED values averaged between sources
Chordeumatida	U	T	Diplopoda - terrestrial unknown	0.349	15725	2, 24	
Polydesmida	U	T	Diplopoda - terrestrial unknown	0.349	15725	2, 24	
Polyzoniida	U	T	Diplopoda - terrestrial unknown	0.349	15725	2, 24	
Spirobolida	U	T	Diplopoda - terrestrial unknown	0.349	15725	2, 24	
**Class: Entognatha**							
Collembola	U	T		0.425	23161	8, 10	pDM from terrestrial insects; ED from AFDW and pASH, 1 species
Collembola	U	A		0.425	23161	8, 10	pDM from terrestrial insects; ED from AFDW and pASH, 1 species
**Class: Insecta**	All	T		0.425	22820	8	Grand means for terrestrial Insects
Archaeognatha	A	T	Insecta - terrestrial all stages	0.425	22820	8, 10	
Coleoptera	A	A		0.29	22472	8, 16	ED value based on one species
Coleoptera	A	T		0.29	23071	8, 16	ED values, separated adults+pupae
Coleoptera	L	A		0.21	21585	9, 16	pDM for combined Coleoptera/Trichoptera; ED from 1 species
Coleoptera	L	T		0.25	23811	8, 25	pDM is average value; ED values separated larvae/nymphs
Coleoptera	L	U		0.23	22698	8, 9, 16, 25	pDM and ED values averaged between 2 sources
Dermaptera	All	T		0.425	26800	10, 24	pDM from terrestrial insects; ED adult/larvae pooled
Dermaptera	A	T	Dermaptera - terrestrial all stages	0.425	26800	10, 24	
Diptera	A	T		0.18	23346	2, 16	pDM from winged insects combined; ED value averaged
Diptera	L	A		0.13	22694	8, 16	pDM from aquatic nymphs combined; ED from Chironomidae larvae/pupae
Diptera	L	T		0.227	24196	8, 25	
Diptera	L	U	Diptera - aquatic larvae	0.13	22694	8, 16	
Diptera	P	A	Diptera - aquatic larvae	0.13	22694	8, 16	
Diptera	P	T	Dipera - terrestrial larvae	0.227	24196	8, 25	
Ephemeroptera	A	A		0.18	21600	16, 22	pDM from winged insects combined
Ephemeroptera	A	T		0.18	23060	9, 16	pDM from winged insects combined; ED based on Callibaetis
Ephemeroptera	L	A		0.13	25481	9, 16	pDM from aquatic nymphs combined; ED from Callibaetis
Ephemeroptera	U	A		0.13	25481	9, 16	*aquatic nymphs combined; ED value based on Callibaetis
Ephemeroptera	U	T		0.18	23060	9, 16	*winged insects combined; ED based on Callibaetis
Hemiptera	A	A		0.227	21547	9	averaged values
Hemiptera	A	T	Heteroptera (pDM)	0.482	24415	2, 8	ED value averaged between 2 sources
Hemiptera	L	A	Hemiptera - aquatic adult	0.227	21547	9	

Order	Stage	Habitat	Surrogate	pDM	ED	Ref	Notes
Hemiptera	L	T	Hemiptera - terrestrial adult	0.482	24415	2, 8	
Hemiptera	L	U	Hemiptera - aquatic adult	0.227	21547	9	
Hemiptera	U	T	Hemiptera - terrestrial adult	0.482	24415	2, 8	
Hymenoptera	A	T		0.24	22403	2, 16, 22	ED value averaged between 2 sources
Hymenoptera	L	T	Tenthredinoidea	0.193	23110	2	
Isoptera	A	T	Insecta - terrestrial all stages	0.425	22820	8, 10	
Lepidoptera	A	T		0.5	21810	2, 24	ED value averaged between 2 sources
Lepidoptera	L	A	Lepidoptera - terrestrial larva	0.25	22725	2, 24	
Lepidoptera	L	T		0.25	22725	2, 24	ED value averaged between 2 sources
Lepidoptera	P	T	Lepidoptera - terrestrial larva	0.25	22725	2, 24	
Megaloptera	A	T	Megaloptera - aquaitc larva	0.18	21719	8, 16	pDM for winged insects combined
Megaloptera	L	A		0.13	21719	8, 16	pDM for aquatic nymphs; ED based on one species
Neuroptera	A	T		0.18	20400	16, 22	pDM for winged insects combined; ED based on aquatic
Neuroptera	L	T		0.229	26800	24, 25	pDM avg. of larval Homoptera, Lepidoptera, Diptera, Coleoptera
Neuroptera	U	T	Neuroptera - terrestrial larva	0.229	26800	24, 25	
Odonata	L	A			24600	5	
Orthoptera	All	T		0.22	22175	8, 16	ED value averaged across various stages
Orthoptera	A	T	Orthoptera - terrestrial all stages	0.22	22175	8, 16	
Orthoptera	L	T	Orthoptera - terrestrial all stages	0.229	22175	8, 25	pDM avg. of larval Homoptera, Lepidoptera, Diptera, Coleoptera
Plecoptera	U	U		0.18	23220	2, 16	ED value no stage specified
Plecoptera	A	T	Plecoptera - unknown	0.18	23220	2, 16	pDM for winged insects combined
Plecoptera	L	A	Plecoptera - unknown	0.13	23220	2, 16	pDM for aquatic nymphs combined
Psocoptera	A	T	Psocoptera - unknown	0.297	21650	19	
Psocoptera	L	T	Psocoptera - unknown	0.297	21650	19	
Psocoptera	U	T		0.297	21650	19	Life stage not specified
Raphidioptera	A	T	Insecta - terrestrial all stages	0.425	22820	8, 10	
Thysanoptera	U	T		0.425	26799	10, 18	pDM from terrestrial insects; ED from 1 species, stage unspecified
Thysanoptera	A	T	Thysanoptera - terrestrial unknown	0.425	26799	10, 18	
Thysanoptera	L	T	Thysanoptera - terrestrial unknown	0.425	26799	8, 10	
Trichoptera	A	A		0.18	21600	16, 22	pDM for winged insects combined
Trichoptera	A	T		0.18	24009	9, 16	pDM for winged insects combined; ED based on one species
Trichoptera	L	A		0.21	20986	8, 16	pDM for Coleoptera and Trichoptera combined; ED for various larvae/nymph stages
Trichoptera	P	A	Trichoptera - aquatic larva	0.21	20986	8, 16	
Trichoptera	P	T	Trichoptera - aquatic larva	0.21	20986	8, 16	

Order	Stage	Habitat	Surrogate	pDM	ED	Ref	Notes
**Class: Malacostraca**							
Amphipoda	I	A	Amphipoda - aquatic unknown	0.208	16744	8, 9	pDM value is averaged between sources
Amphipoda	U	A		0.208	16744	8, 9	
Amphipoda	U	A		0.208	16744	8, 9	pDM value is averaged between sources; ED from Source 8
Isopoda	U	A		0.25	12569	8	Based on one species
Isopoda	U	A		0.25	12569	8	Based on one species
Mysida	U	A	Amphipoda for Mysida	0.208	16744	8, 9	pDM value averaged between sources; ED from Source 8
	U	A	Cladocera and Copepoda	0.061	24121	8, 15	ED average value from Source 8; pDM averaged between sources
**Phylum: Chordata**	U	A	Salmoniformes - aquatic immature	0.263	24037	3, 4	Assumed to be trout fry in our study
	Egg	A		0.414	15054	8	Values from mature (unfertilized) eggs from Salmonidae
**Class: Actinopterygii**							
Salmoniformes	I	A		0.263	24037	3, 4	ED value based on rainbow trout
Scorpaeniformes	A	A		0.231	21737	8, 20	pDM from slimy and deepwater sculpin; ED average of 2 sources
Scorpaeniformes	I	A		0.231	21737	8, 20	pDM from slimy and deepwater sculpin; ED average of 2 sources
**Class: Amphibia**							
Anura	L	A		0.095	12772	12	ED converted from wet weight DW using water content reported
**Class: Hyperoartia**							
Petromyzontiformes	L	A		0.217	25307	6, 13	ED and pDM values are averaged between sources
**Phylum: Cnidaria**							
Hydrozoa	U	A		0.011	23200	14, 23	pDM from Limnomedusa; ED from 1 species of cultured pop.
**Phylum: Cnidaria**							
**Class: Bivalvia**							
Veneroida	L	A		0.915	228.6	10	Based on two species
**Phylum: Mollusca**							
**Class: Gastropoda**							
Planorbidae	U	A	Planorbidae - aquatic unknown	0.538	7139	8	ED based on AFDM; biomass equations based on dry weight without shell, but ED values included shell
Basommatophora	L	A	Planorbidae - aquatic unknown	0.538	7139	8	
Basommatophora	I	A	Planorbidae - aquatic unknown	0.538	7139	8	
Basommatophora	U	A	Planorbidae - aquatic unknown	0.538	7139	8	
	I	T	Planorbidae - aquatic unknown	0.538	7139	8	
	U	T	Planorbidae - aquatic unknown	0.538	7139	8	
Heterostropha	U	A	Planorbidae - aquatic unknown	0.538	7139	8	
Neotaenioglossa	U	A		0.538	1937	8	ED based on DW including ash; biomass regression included shell
Stylommatophora	U	T	Planorbidae - aquatic unknown	0.538	7139	8	

Order	Stage	Habitat	Surrogate	pDM	ED	Ref	Notes
**Phylum: Nematoda**							
Nemata	U	A	Nematoda - aquatic unknown	0.25	21317	7, 11, 17	
Trichurida	A	T	Nematoda - aquatic unknown	0.25	21317	7, 11, 17	
Nematoda	U	A		0.25	21317	7, 11, 17	pDM approximation from 2 sources; ED is averaged value
**Phylum: Nematomorpha**	U	A	Nematoda - aquatic unknown	0.25	21317	7, 11, 17	
**Phylum: Platyhelminthes**	U	A		0.18	21840	7, 25	Based on one species
Tricladida	U	A	Turbellaria - aquatic unknown	0.18	21840	7, 25	

References

1. Albert, A.M. 1983. Energy budgets for populations of long-lived arthropod predators (Chilopoda: Lithobiidae) in an old beech forest. Oecologia 56:292-305.
2. Broadman, P.A., and Reyer, H.U. 1999. Nestling provisioning in water pipits (*Anthus spinoletta*): do parents go for specific nutrients or profitable prey? Oecologia 120:506-514.
3. Ciancio, J., and Pascual, M. Energy density of freshwater Patagonian organisms. Ecología Austral. 2006;16(01):091-4.
4. Ciancio J.E., Pascual, M.A., and Beauchamp, D.A. Energy density of Patagonian aquatic organisms and empirical predictions based on water content. Transactions of the American Fisheries Society 136:1415-22.
5. Clarke, A., Prince, P.A. and Clarke, R. 1996. The energy content of dragonflies (Odonata) in relation to predation by falcons. Bird Study, *43*:300-304.
6. Cochran, P.A., Hodgson, J.Y., and Kinzinger, A.P. 2003. Change in energy density of the sea lamprey (*Petromyzon marinus*) during its parasitic phase: implications for modeling food consumption and growth. *Journal of Great Lakes Research*, *29*:297-306.
7. Croll, N.A., and de Soyza, K. 1980. Comparative calorie values of nematodes. Journal of Nematology 12:132.
8. Cummins, K.W., and Wuycheck, J.C. 1971. Caloric equivalents for investigations in ecological energetics. International Association of Theoretical and Applied Limnology 18:1-158.
9. Driver, E.A., Sugden, L.G., and R.J. Kovach. 1974. Calorific, chemical and physical values of potential duck foods. Freshwater Biology 4:281-292.
10. Eggleton, M.A., and Schramm, H.L. 2004. Feeding ecology and energetic relationships with habitat of blue catfish, *Ictalurus furcatus*, and flathead catfish, *Pylodictis olivaris*, in the lower Mississippi River, USA. Environmental Biology of Fishes 70:107-121.
11. Ellenby, C. 1968. Determination of the water content of nematode worms by interference microscopy. Experientia 24:84-85.
12. Evenson, E.J., and Kruse, K.C. 1982. Effects of a diet of bullfrog larvae on the growth of Largemouth Bass. The Progressive Fish-Culturist 44:44-46.
13. Farmer, G.J. 1974. Food consumption, growth, and host preferences of the sea lamprey, *Petromyzon marinus* L., Ph.D. Dissertation, University of Guelph, Guelph, Ontario.
14. Jankowski, T., 2000. Chemical composition and biomass parameters of a population of *Craspedacusta sowerbii* Lank 1880 (Cnidaria: Limnomedusa). *Journal of Plankton Research*, *22*:1329-1340.
15. Luecke, C., and Brandt, D. 1993. Estimating the energy density of daphnid prey for use with rainbow trout bioenergetics models. Transactions of the American Fisheries Society 122:386-389.
16. McCarthy, S.G., Duda, J.J., Emlen, J.M., Hodgson, G.R. and Beauchamp, D.A. 2009. Linking habitat quality with trophic performance of steelhead along forest gradients in the South Fork Trinity River watershed, California. Transactions of the American Fisheries Society 138:506-521.
17. Menti, H., Wright, D.J. and Perry, R.N. 1997. Desiccation survival of populations of the entomopathogenic nematodes *Steinernema feltiae* and *Heterorhabditis megidis* from Greece and the UK. Journal of Helminthology 71:41-46.
18. Naess, S.J., Steigen, A.L. and Solhøy, T., 1975. Standing crop and calorific content in invertebrates from Hardangervidda. In *Fennoscandian Tundra Ecosystems*. Springer, Berlin, Heidelberg. pp. 151-159.
19. Norberg, R.Å. 1978. Energy content of some spiders and insects on branches of spruce (*Picea abies*) in winter; prey of certain passerine birds. Oikos 31:222-229.

20. Rottiers, D.V., and Tucker, R.M. 1982. Proximate composition and caloric content of eight Lake Michigan fishes. Technical Ppaers of the Fish and Wildlife Service Vol. 108, US Department of the Interior, Fish and Wildlife Service, Washington, D.C.
21. Salmon, S.J., 1973. Studies on the ecology and energetics of *Neobisium muscorum* (Leach) and *Chthonius orthodactylus* (Leach)(Pseudoscorpiones: Arachnida). PhD. Dissertation, University of Leichester, UK. https://ethos.bl.uk/OrderDetails.do?uin=uk.bl.ethos.737216.
22. Sato, T., Watanabe, K., Kanaiwa, M., Niizuma, Y., Harada, Y. and Lafferty, K.D. 2011. Nematomorph parasites drive energy flow through a riparian ecosystem. *Ecology 92*:201-207.
23. Schroeder, L.A., and Callaghan, W.M. 1982. Effects of temperature on the energy budgets of *Hydra pseudoligactis*. Oecologia, 53:238-244.
24. Török, J., and Ludvig, E. 1988. Seasonal changes in foraging strategies of nesting blackbirds (*Turdus merula* L.). Behavioral Ecology and Sociobiology 22:329-333.
25. Tsukamoto, J. 1988. Dry/preserved wet weight ratio of soil macro-animals in forests. Ecological Research *3*:61-65.
