# Supplementary material for: Shifting food web structure during dam removal—Disturbance and recovery during a major restoration action
Source: PLoS One. 2020 Sep 29;15(9):e0239198. doi: 10.1371/journal.pone.0239198 (PMC7523948; doi:10.1371/journal.pone.0239198)
Supplement: Attachment — Submitted filename: Review.docx [file pone.0239198.s009.docx]

**General Comments**

This manuscript contains a massive amount of information and provides a comprehensive picture of some of the impacts of sedimentation during dam-removal on invertebrate prey availability and salmonid diets. I do think it needs to be edited further. See detailed comments below for some examples.

Furthermore, given the amount of information presented, any streamlining would make the paper easier to read. This paper is unconventional in its length and breadth. This is not necessarily a bad thing. A more conventional treatment might be to split this into multiple papers and focus the story on a specific aspect of the larger story in each. I will not say that either approach is superior, and certainly there are advantages to both. This broad style of paper provides all the relevant information in one spot, and supports big picture thinking, which is important in ecology and arguably we need to do more of it. However, a shorter paper is faster and easier to process when considering one aspect of the bigger story. It may also be possible to have multiple shorter papers that build upon one another. It may be possible to remove electivity, CPUE, and condition index, and still tell much of the current story.

If the current breadth is kept, I think that the introduction needs to better set the stage for what will be in the paper. There is some detailed analysis in this paper about electivity and the like, which are never mentioned in the introduction. See comment below.

Lastly, it is impossible to tell if the data are available, because not enough detail is given to locate them. More information is needed on the location of the data beyond <https://www.sciencebase.gov/catalog/> and <https://www.data.gov/>

**Detailed Comments**

Could background on Elwha Dam removal be condensed?

Line 131 replace changed with varied?

Because the paper covers so many topics (e.g., prey availability, diet, selectivity) It might be nice to set the stage in the introduction for why these matter and how they are all related. As is, intro is focused on background on dam removal and sediment, without much discussion of biology. Makes it a bit surprising when things like electivity, CPUE, and condition factor are brought in during the methods.

216 Were velocities measured at the beginning and end of the sample period?

254 perhaps change “sample metrics” to something more informative. You talk about statistical analysis in this section too.

261 “section 3.4” ?

268-277 Not a critique on scientific soundness, but this paper is already quite complex and large. Is adding CPUE and condition important for the overall story. Should it be mentioned in the introduction?

271. is CPUE in the river meaningful if no consideration for effort (in terms of time) other than the 90 minutes max effort. Maybe it is if it was rare to get the 10 fish within the 90 minutes.

282. does it make sense to include empty stomaches? Potentially conflating diet composition similarity, and “fulness” similarity. The fish with empty stomachs are eating something, just not when they were sampled. Therefore, I would probably just exclude the empty stomachs form the analysis.

278-284, assessing similarity of diet and ambient prey, and selectivity, was not mentioned in objectives.

285, index of relative *Importance* not *Abundance*

289 I believe this is different than the traditional IRI from Hart et al 2002, which is F(N+V), where F = frequency of occurrence percentage, N = numerical percentage, and V = volumetric percentage. But, I’m not sure what the “mean relative numeric abundance of order i” or “mean relative energetic abundance of order i” are. Maybe they are the same as percentages? If they are different, it would require some explanation for why the modification was made. Also, is %F frequency of occurrence percentage, or just frequency of occurrence, as written?

295 Hart et al 2002 sat that IRI should not be compared across groups, but rather ranks of different prey types should be compared. The method of standardizing IRI and then comparing may be valid, but there should be a citation to support it or some explanation for why it is valid. Since I can’t really tell what the calculated IRI is, I definitely can’t tell if this method of comparing across groups is valid.

296-298 No citation indicates that you developed this method of evaluating selectivity? It seems like it might be valid, but there are other more established electivity indices.

306 What was the critical value used for Tukeys HSD test?

313 How were age classes determined? sample sizes?

317 why are “section” and “year” capitalized

345 where do energy density values come from in table 2?

416. of course things “changed” in the reference section in some way or another. In some instances, it looks like there was more change in the control than in the sediment impacted sections. More detail would help. Is this referring to statistical significance?

427. Except summer ME increased.

496 consider changing “prey availability” to “energy availability”

545 Figure 11. Perhaps mention that a couple stress values are >.2

622 what do the different letters represent?

809 might these smolt data be a better indication of fish densities than your river % CPUE?

820 could turbidity reduce predation risk and therefore increase the duration or locations where salmonids can “safely” forage?

819 any “pink salmon year” effect?

829 5.3.1 sediment?
