# Supplementary material for: Shifting food web structure during dam removal—Disturbance and recovery during a major restoration action
Source: PLoS One. 2020 Sep 29;15(9):e0239198. doi: 10.1371/journal.pone.0239198 (PMC7523948; doi:10.1371/journal.pone.0239198)
Supplement: Attachment — Submitted filename: Response to Reviewers.docx [file pone.0239198.s010.docx]

Dear Dr. Doi,	08/08/2020

Summarized in the table below are our responses to reviewer comments from initial review of PONE-D-20-06632: Shifting food web structure during dam removal—disturbance and recovery during a major restoration action. We have included a similar table of comments made during the USGS internal review process so it clear why additional (small) changes were made in the manuscript.

Thanks you for the opportunity to resubmit the manuscript.

Respectfully,

Sarah Morley

| **REVIEWER COMMENTS - PLOS ONE** | **RESPONSE** |  |
| --- | --- | --- |
| **General** |  |  |
| This manuscript contains a massive amount of information and provides a comprehensive picture of some of the impacts of sedimentation during dam-removal on invertebrate prey availability and salmonid diets. I do think it needs to be edited further. Given the amount of information presented, any streamlining would make the paper easier to read. This paper is unconventional in its length and breadth. This is not necessarily a bad thing. A more conventional treatment might be to split this into multiple papers and focus the story on a specific aspect of the larger story in each. I will not say that either approach is superior, and certainly there are advantages to both. This broad style of paper provides all the relevant information in one spot, and supports big picture thinking, which is important in ecology and arguably we need to do more of it. However, a shorter paper is faster and easier to process when considering one aspect of the bigger story. It may also be possible to have multiple shorter papers that build upon one another. It may be possible to remove electivity, CPUE, and condition index, and still tell much of the current story. | We agree that this manuscript contains a large amount of data and is quite long, but our goal is to provide a more comprehensive picture that supports big picture thinking. Selectivity is a big piece of the story that helps tie prey availability and diet composition together and we think it is important to retain. However we have removed all text and tables and figures relating to CPUE and condition factor as suggested. This coupled with streamlining of text (particularly in the Introduction) has reduced the overall length of the paper by ~ 10%. To reduce the total number of figures in the paper, we have moved the four shade plots to Supplemental Materials. |  |
| It is impossible to tell if the data are available, because not enough detail is given to locate them. More information is needed on the location of the data beyond <https://www.sciencebase.gov/catalog/> and <https://www.data.gov/> | We have updated this information in the acknowledgement section and added formal citations for both the estuary and river datasets:  Data generated in this study and associated efforts monitoring environmental metrics before and during Elwha dam removal are available at <https://doi.org/10.5066/F75B00N4> and at <https://doi.org/10.25923/eg1s-t677> |  |
| **Introduction** |  |  |
| If the current breadth is kept, I think that the introduction needs to better set the stage for what will be in the paper. There is some detailed analysis in this paper about electivity and the like, which are never mentioned in the introduction. | We have added additional information about what we measured in the final paragraph of the introduction and specifically mention selectivity. |  |
| Because the paper covers so many topics (e.g.,  prey availability, diet, selectivity) It might be nice to set the stage in the introduction for why these matter and how they are all related. As is, intro is focused on background on dam removal and sediment, without much discussion of biology. Makes it a bit surprising when things like electivity, CPUE, and condition factor are brought in during the methods. | Good point. We have added the following sentence in the final paragraph of the introduction: “Because the restoration of anadromous fish communities was a primary goal of the project, it is important to understand how the short-term effects of dam removal impacted the prey base, selectivity, and diet of juvenile salmon.” |  |
| Could background on Elwha Dam removal be condensed? | We feel it is important to keep the details on the timeline of sediment impacts during dam removal to set the stage for the biological results that follow, but have significantly streamlined dam removal background by reducing text ~ 25%. |  |
| Line 131 replace changed with varied? | We think “changed” is the appropriate word choice in this context as it refers to shifts in prey availability over time and space, rather than simply describing natural variability. |  |
| **Methods** |  |  |
| 216 Were velocities measured at the beginning and end of the sample period? | No, velocity was only measured at the beginning of the sample period. Measurements were taken at four locations relative to net placement in order to accurately calculate total sample volume. As the typical sample period was only 30 minutes (up to a max of 60), we do not expect that velocity changed much (if at all) during that interval. |  |
| 254 perhaps change “sample metrics” to something more informative. You talk about statistical analysis in this section too. | We think “Sample Metrics” is the appropriate term to describe the specific variables we measured, but have removed any text describing statistical analyses from this section. |  |
| 261 “section 3.4” ? | This text has been removed. |  |
| 268-277 Not a critique on scientific soundness, but this paper is already quite complex and large. Is adding CPUE and condition important for the overall story. Should it be mentioned in the introduction? | Agreed. We have removed all text related to CPUE and condition factor from this paper. |  |
| 271. is CPUE in the river meaningful if no consideration for effort (in terms of time) other than the 90 minutes max effort. Maybe it is if it was rare to get the 10 fish within the 90 minutes. | No longer relevant, we have removed CPUE from the paper. |  |
| 282. does it make sense to include empty stomaches? Potentially conflating diet composition similarity, and “fulness” similarity. The fish with empty stomachs are eating something, just not when they were sampled. Therefore, I would probably just exclude the empty stomachs form the analysis. | Due to the way in which %IRI is calculated, fish with empty stomachs do not alter these calculations. To avoid confusion, we have moved the sentence regarding the dummy variable to the permanova section under “Statistical analyses”. |  |
| 278-284, assessing similarity of diet and ambient prey, and selectivity, was not mentioned in objectives. | We have added text to the introduction expanding objectives to selectivity |  |
| 285, index of relative *Importance* not *Abundance* | Corrected |  |
| 289 I believe this is different than the traditional IRI from Hart et al 2002, which is F(N+V), where F = frequency of occurrence percentage, N = numerical percentage, and V = volumetric percentage. But, I’m not sure what the “mean relative numeric abundance of order i” or “mean relative energetic abundance of order i” are. Maybe they are the same as percentages? If they are different, it would require some explanation for why the modification was made. Also, is %F frequency of occurrence percentage, or just frequency of occurrence, as written? | We changed our wording to conform with how IRI calculations are described in the original papers. We have replaced “relative abundance” with “percentage”. Our approach is very similar to that described by Hart et al. 2002, but modified in the following ways:  -we use energetic content (J for joules) rather than volumetric (V for volume) content of prey. Energy is more relevant than volume in terms of prey quality, as alluded to by Pinkas et al. 1971 in the original description of IRI.  -we make these calculations at the order rather than species level (hence the addition of the word “mean” in parameter definitions).  F is frequency of occurrence percentage |  |
| 295 Hart et al 2002 sat that IRI should not be compared across groups, but rather ranks of different prey types should be compared. The method of standardizing IRI and then comparing may be valid, but there should be a citation to support it or some explanation for why it is valid. Since I can’t really tell what the calculated IRI is, I definitely can’t tell if this method of comparing across groups is valid. | Hart et al. 2002 goes on to state “Two recent reviews of dietary assessments in fisheries biology both recommended the IRI as a valuable alternative to single index measures, while expressing it as a percentage facilitated comparisons between different samples (Cortes 1997; Liao et al. 2001). “ |  |
| 296-298 No citation indicates that you developed this method of evaluating selectivity? It seems like it might be valid, but there are other more established electivity indices. | We explored a number of different selectivity indices, but every index has limitations and biases. For instance, some are based only on relative numeric abundance and thus do not account for frequency of occurrence or caloric value. Others are better designed to detect changes in fish behavior that are not related to changes in food density. While the %IRI approach we use is not perfect either, we feel it is more comprehensive in capturing frequency as well as numeric, and energetic abundances. This approach also generated plots that allowed us to concisely display changes over time for all taxa instead of focusing on only a handful. The response observed in our data was really about shifts over many different groups, rather than driven by just a few taxa. |  |
| 306 What was the critical value used for Tukeys HSD test? | Q values varied depending on the sample size of the test population, but we inserted a statement re alpha value. |  |
| 313 How were age classes determined? sample sizes? | Age classes were based on previous studies of spawn timing and emergence in the Elwha, and histograms of forklength. |  |
| 317 why are “section” and “year” capitalized | Corrected |  |
| **Results** |  |  |
| 345 where do energy density values come from in table 2? | As described in the last paragraph under “Data collection”: energy density values were calculated based on body parameter measurements we collected from each invertebrate and the regression equations presented in Tables S3 and S4 which convert body parameter measurements to biomass and energy. |  |
| 416. of course things “changed” in the reference section in some way or another. In some instances, it looks like there was more change in the control than in the sediment impacted sections. More detail would help. Is this referring to statistical significance? | Yes, this refers to statistical significance for the sample metrics evaluated in this study. We have added the word “significant” to this sentence. |  |
| 427. Except summer ME increased. | The increase in ME was not statistically significant. We changed the sentence to read: “Drift density and taxa richness declined in **most** impacted sections during dam removal…” |  |
| 496 consider changing “prey availability” to “energy availability” | Changed |  |
| 545 Figure 11. Perhaps mention that a couple stress values are >.2 | We added the following caveat to the caption for Figures 3, 7, and 8 “Note that ordinations with 2D stress values > 0.2 may not adequately represent data structure (Clarke et al. 2014).” |  |
| 622 what do the different letters represent? | Significance of pairwise tests; as indicated in table footnotes. |  |
| **Discussion** |  |  |
| 809 might these smolt data be a better indication of fish densities than your river % CPUE? | We have removed this text |  |
| 820 could turbidity reduce predation risk and therefore increase the duration or locations where salmonids can “safely” forage? | Perhaps, although the reduced growth and survival shown in many experimental and field studies suggest that such benefits are outweighed by the negative impacts. If turbidity is high enough to reduce predation risk, it is also likely high enough to impede foraging. |  |
| 819 any “pink salmon year” effect? | We have removed this paragraph. Pink returns to the Elwha before and during dam removal were very low. |  |
| 829 5.3.1 sediment? | Removed |  |
| **Additional changes not specifically requested by reviewer:** | |  |
| References | We have updated references to comply with the PLOS numbered citation method. |  |
| Figures | We have improved image resolution on all Figures |  |
|  | Due to the high number of figures in the manuscript, we moved four of the figures (shade plots) into supplemental materials. |  |

| **REVIEWER COMMENT - USGS INTERNAL** | **RESPONSE** |
| --- | --- |
| **Abstract** |  |
| Line 31. We found that sediment impacts from dam removal on the Elwha River significantly reduced invertebrate prey availability, but juvenile salmon adjusted their foraging so that the amount of energy in diets was similar before and after[RSP1] dam removal. | [RSP1]During?  Corrected |
| Line 43. As we move further[RSP1] along the dam removal response trajectory, we hypothesize that food web complexity will continue to increase as the annual sediment load approaches natural background levels, as anadromous fish recolonize the majority of the watershed between and above the former dams, and as revegetation and microhabitats continue to develop in the estuary. | [RSP1]Farther?  Farther is typically used to refer to physical distances and further for figurative distances. Retaining further. |
| **Methods** |  |
| Line 157. Hatchery Chinook are not externally marked, nor are 100% of [RSP1] hatchery coho. | [RSP1]What’s this about? Maybe that unlike Chinook, coho aren’t externally or internally marked? Rephrasing needed?  We have removed this sentence as hatchery influence is no longer discussed in the discussion section with the removal of fish density and condition factor from the paper. |
| Line 284. To visualize shifts in prey selectivity over time, we compared invertebrate prey availability to diet composition using a modified Index of Relative Abundance[RSP1] (IRI) approach (Hart et al. 2002). | [RSP1]Importance? If not, why not IRA?  Corrected |
| **Results** |  |
| Line 341. Amphipods and ostracods [RSP1] were among the most abundant taxa in benthic samples prior to dam removal, but were completely absent following dam removal (Figs 3b and 4b). | [RSP1]You lumped over these taxa in the stacked bar figures. Are they in the Aquatic (misc) category?  We have clarified text to state “(see Aquatic (Misc) category in Figs 3b and 4b).” |
| Line 507. Estuarine Chinook and coho selected for lavage sampling were between 45 to 195 mm in length (mean = 96.7 mm, SD = 22.1 mm) and mass ranged from 0.9 g to 55 g (mean = 8.5 g, SD = 6.5 g). | [RSP1]Maybe give overall percent Chinook versus coho across all that were lavaged?  We added the sentence “The relative abundance of the two species varied over the study period. Chinook comprised 32% of diet samples before dam removal versus 30% during dam removal in spring, and 7% before versus 48% during dam removal in summer.” |
| Line 650.Bolded black values indicate *P* ≤ 0.05 for main test and spring pairwise comparisons, and ≤ 0.0083 (Bonferroni adjusted) for summer pairwise comparisons. [RSP1] | [RSP1]Why aren’t any of the p-values for pairwise comparisons bolded? Looks to me like some of them meet your significance criteria.  Corrected |
| **Tables** |  |
| Table 2 | [RSP1]Widen columns so values fit on one line  Corrected |
| **Figures** |  |
| Fig 2. Timeline of Elwha River dam removals. Dates are shown relative to (a) Sampling schedule for fish and invertebrate collections[RSP1] | [RSP1]Move “Estuary sampling” up and “River sampling” down to align correctly with horizontal sampling duration bars?  Done |
| Fig 2. Timeline of Elwha River dam removals. Dates are shown relative to…,and a stage-discharge residual analysis based on Anderson and Konrad (2019) showing how sediment release from dam removal changed bed elevations across a network of river stage recorders operated by Bountry et al (2018) in (d) the Lower and (e) Middle Elwha River.[RSP2] | [RSP2]What do X1-X24 indicate? Different stage recorder locations? Does it matter where they were?  We have added the following sentence to the caption: “The number of each stage recorder increases in an upstream direction (i.e., X1 closest to the river’s mouth).” |
| Fig 5. Shade plot of estuary invertebrate density over time. Taxa are shown for benthic core (a,b) and fallout samples (c,d) that contributed most to differences between years in spring (a,c) and summer (b,d)[RSP1] , based on the SIMPER routine in PRIMER (≥ 2% dissimilarity) . Mean numerical density is fourth root transformed. | [RSP1]You’ve already labeled Benthic versus Fallout on the figure (as opposed to caption) so why not also label Spring versus Fall (i.e., Benthic, Spring; Benthic, Fall; Fallout, Spring; Fallout, Fall). Also, are taxa listed top-to-bottom in order of %contribution? If so maybe you should state that in the caption? Now that I’ve looked at Figure 7 I’m guessing taxa are not ordered by %contribution but rather by a taxonomic structure of some kind? With insects grouped together and then alphabetical?  We have redone all shade plots to include season in sub-headings. Taxa are grouped by phylum and class - although we have omitted these sub-headings to reduce total figure space. Within a given order, families are listed alphabetically. Due to the high number of figures in the manuscript, we have moved all shade plots to Supplemental Materials. |
| Fig 7. Shade plot of river invertebrate density over time. Taxa are shown that contributed the most to differences between years in (a) spring and (b) summer river drift, based on the SIMPER routine in PRIMER (≥ 2% dissimilarity[RSP1] ). Mean numerical density by river section and year are square-root transformed. | [RSP1]In summer, >2% contribution in LE *and* ME, or >2% contribution in LE *or* ME.  LE **or** ME |
| **Supplemental** |  |
| S1 Table | [RSP1]In S1 Table, habitat column headings (e.g., EL) don’t match table caption (e.g., LE)  Corrected |
